# Small‐Scale Big Science: From Nano‐ to Atomically Dispersed Catalytic Materials

**DOI:** 10.1002/smsc.202200036

**Published:** 2022-10-13

**Authors:** Ligang Wang, Huan Liu, Jiahao Zhuang, Dingsheng Wang

**Affiliations:** ^1^ Department of Chemistry Tsinghua University Beijing 100084 China

**Keywords:** atomically dispersed, catalytic activity, clusters, nanomaterials, sub-nano

## Abstract

The emergence of nanomaterials (NMs) has made everyone more aware of the close relationship between the size and performance of materials. Especially, the catalytic properties of materials may change dramatically when the macroscopic size of matter goes from bulk to nanometer to atomic level. It is very firmly believed that “Small size determines macroproperties, contains big science.” Therefore, as chemists hope, it is very essential to rationally tune and characterize the size of materials and deeply understand the relationship between macroscopic catalytic properties and small structures. Herein, the recent progress in both multi‐scale materials from nanoscale, sub‐nano, clusters to atomic scale for various catalytic reactions is focused. Specifically, the catalytic reaction on the microstructures and the relationship between the small scale and big science are comprehensively summarized and discussed. This review systematically spans dimensions to reveal the catalytic performances of materials at different scales with a unique effect. This is crucial for rationally understanding the composition of materials and exploring their catalytic activity. Current opportunities, future challenges, and outlooks for the application of multiscale materials (NMs, sub‐nano, cluster, and atomically dispersed materials) in catalysis, energy and environmental protection, etc. are summarized and outlined.

## Graphical abstract

The size of a material significantly determines its catalytic performance. Multi‐scale materials (nanomaterials (NMs), sub‐nano, cluster, and atomically dispersed materials) with unique effects, such as size effect confinement effect, defect effect, strain effect, metal–support interactions effect, and coordination effect in catalysis such as electrocatalysis, photocatalysis, and thermocatalysis are systematically discussed.

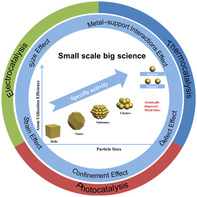

## 1 Introduction

The development of human civilization has continuously added new branches to the evolutionary tree of materials, and new members have been added to the list of traditional materials today, even though they also had the status of new materials at the beginning. From mud bricks and wood, to copper, iron, aluminum, and steel, to carbon fiber and graphene, with the progress of civilization and science and technology, the breadth and depth of human development and utilization of materials have gradually deepened. Nowadays, more and more new materials have entered the field of the vision of scientists. The breakthrough of new material technology will be accompanied by a new round of industrial revolution, changing the attributes of the world we live in. There is reason to believe that any grand project of human beings in the future will be supported by seemingly “small” new materials, and we should see them everywhere in our daily life. Nowadays, with the development of human knowledge reserves and science and technology, their understanding of materials has gone deep into the macroscopic celestial bodies and microscopic particle levels. The size of a material is inextricably linked to its properties. The objective material world can be divided into multiple levels according to the scale, such as nano, sub‐nano, cluster, and atomic levels.

In the 1960s, R.P. Feynman, a famous American physicist and Nobel Prize winner, first proposed the idea of the basic concept of “nanotechnology.” This idea came from a report he gave at the American Physical Congress in 1959, titled “there is plenty of room at the bottom.”^[^ ^1^ ^]^ The object of “nanochemistry” research is the chemical entity with a size of 1–100 nm, which constitutes a mesoscopic phase between the microscopic phase and the macroscopic phase.^[^ ^2^ ^]^ The mesoscopic phase not only reflects the transition of chemical entities from microscopic to macroscopic in size, but also exhibits a series of special effects and functions.^[^ ^3^ ^]^ Subsequently, the rapid development of nanomaterials (NMs) and nanotechnology has driven the field of materials to the forefront. In 2010, physicists Andre Geim and Kostya Novoselov of the University of Manchester won the Nobel Prize in Physics for separating graphene from graphite, which promoted the rapid development of 2D NMs.^[^ ^4^ ^]^ Graphene is currently one of the most concerning new materials. Its super toughness, electrical conductivity, thermal conductivity, and light transmittance make it have huge development space and can be widely used in electronics, aerospace, biomedicine, and other fields, and its commercial application will drive a series of industries such as information, energy, and biology to change.

With the deepening understanding of researchers for NMs and the development of advanced electron microscopy characterization techniques, research has moved toward cluster science with a size smaller than nanometers. Clusters are relatively stable microscopic or sub‐microscopic aggregates composed of several or even thousands of atoms, molecules, or ions through physical or chemical binding forces, and their physical and chemical properties vary with the number of atoms contained.^[^ ^5^ ^]^ The spatial scale of clusters is in the range of several angstroms to hundreds of angstroms, which is too large to describe with inorganic molecules, and too small to describe with small solids. Many properties are different from single atoms and molecules, as well as solids and liquids. Therefore, people regard clusters as a new level of material structure among atoms, molecules, and macroscopic solid matter, a transition state of various substances from atoms and molecules to bulk matter, or, in other words, representing a condensed state the initial state of matter.

**Figure 1:**
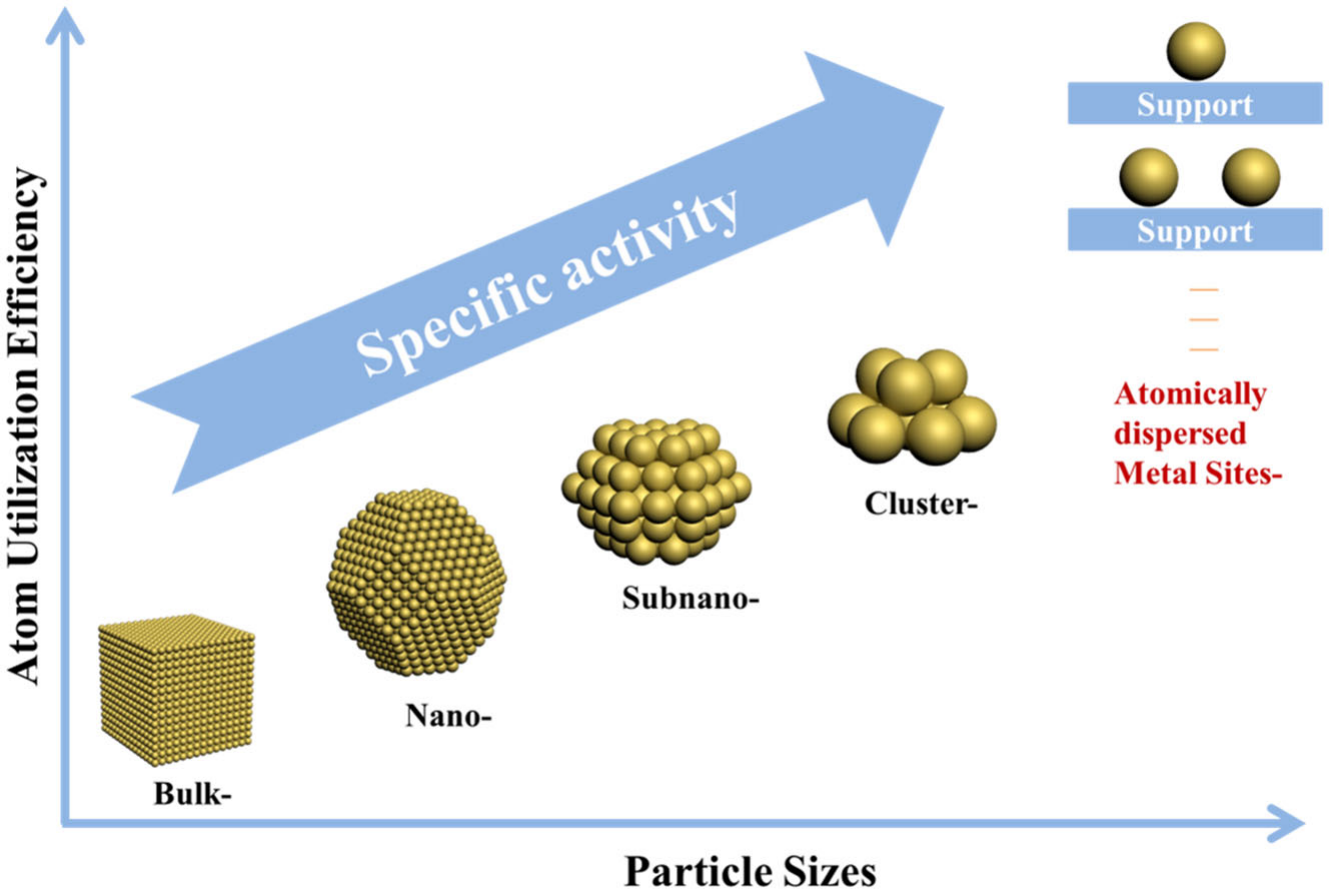
Schematic diagram of the research on multi‐scale and corresponding activity presented in this review.

Benefiting from the characterization techniques development of spherical aberration‐corrected transmission electron microscopy (TEM) and synchrotron‐radiation X‐ray absorption fine structure (XAFS), the single‐atom materials emerged as the times demand and clearly was confirmed, which helped push materials research from “nano‐scale” to “atomic scale” step by step.^[^ ^6^ ^]^ In 2011, Zhang and co‐workers formally established the concept of single‐atom catalysts (SACs) and the basic techniques to study the SACs.^[^ ^7^ ^]^ SACs are different from nano‐ and sub‐nanocatalysts. Because when the particle dispersion reaches the single‐atom size, many new properties are induced, such as the sharply increased surface free energy, quantum size effect, unsaturated coordination environment, and metal–support interaction.^[^ ^8^ ^]^ It is these properties that are significantly different from nano‐ or sub‐nano‐scale particles that endow SACs with their superior catalytic performance. SACs greatly improve the utilization efficiency of metal atoms while ensuring the polar metal loading; they can change the adsorption/desorption selectivity of active components on the catalyst to different molecules, thereby affecting the reaction kinetics. The biggest challenges in the development of SACs are their low metal atom loading density, uncontrollable polymerization, and ambiguous interaction between metal atoms and supports, making it difficult to effectively enhance their catalytic performance. In this review, we summarize and review the development process of materials from nano‐scale, sub‐nano, clusters to the atomic scale, the existing properties of materials at various scales, and present the major challenges and outlooks for future development of multi‐scale functional materials in terms of between functional modulation, and potential catalytic applications (**Figure** **1**).

## 2 Nanoscale

NMs are materials with at least one dimension between 1 and 100 nm. Since Faraday discovered that the color of Au colloid changes with its particle size in the 1850s, people began to explore and study the particle systems within 1–100 nm tirelessly. In the 1960s, R.P. Feynman predicted that when the size of materials is reduced to the nanometer scale (1–100 nm), their physical and chemical properties would undergo huge changes that cannot be predicted by traditional theories. It is also predicted that people would soon enter the “Nano Era.” Since the 1970s, the research and application of NMs have entered a mode of rapid development. The characterization technologies of various materials have emerged, constantly refreshing the breadth and depth of people's cognition. Naturally, a discipline of 1–100 nm micro‐scale has come into being, which is nanotechnology. Nanotechnology has made great progress and becomes a comprehensive disciplinary system that includes multiple disciplines, multiple fields, and rich connotations. Nowadays, Feynman's prediction has come true, NMs have approached every corner of human production and life, from personal food, clothing, housing, and transportation to human exploration of the ultimate mystery of the universe. The wide application of NMs not only changed people's way of life, but also silently promote social reform. Compared with macroscopic bulk materials, NMs have special properties.^4^, ^9^ Surface effect refers to the ratio of the number of atoms on the surface of a nanoparticle to the total number of atoms in the nanoparticle increases sharply with the decrease of the size of the nanoparticle, which eventually leads to the changes of the physical and chemical properties of the nanoparticle. The small size effect refers to the change of macroscopic physical and chemical properties caused by the reduction of nanoparticle size. The quantum size effect refers to the phenomenon that electron energy level changes from continuous energy level to discrete energy level when particle size decreases to a certain value. Quantum tunneling refers to the fact that when the total energy of a microscopic particle is less than the height of the barrier, the particle can still cross the barrier. Based on the unique properties mentioned earlier, NMs have been widely used in many fields. Dimensionality is a key attribute used to differentiate NMs. Based on the dimension size, NMs can be classified into 0D, 1D, 2D, and 3D NMs.

The 0D NMs usually mean that three dimensions of materials lie in the nanoscale range (1–100 nm), usually including quantum dots (QDs), and nanospheres. Among them, the QDs are important semiconductor materials widely used in lasers, transistors, solar cells, light‐emitting diodes, and photodetectors. The synthesis methods of QDs can be roughly divided into chemical solution growth method, epitaxial growth method, and electric field confinement method. Taking different strategies can produce different kinds of QDs, such as PbS QDs and carbon quantum dots (CQDs).

The 1D NMs demonstrate that two dimensions are in the vicinity of the nanoscale, but the last one is not. The 1D structure units in NMs can be divided into nanowires (NWs), nanorods (NRs), nanotubes (NTs), and nanoribbons. One of the typical representatives of NTs is carbon nanotubes (CNTs). The 1D CNTs structure is to seamlessly curl the graphite layer at a certain helical angle. CNTs exhibit good electrical and mechanical properties due to their special structure. In addition, CNTs can often be prepared by chemical vapor deposition (CVD) and arc discharge methods. The growth conditions are adjusted during the preparation process to obtain CNTs with different wall layers. The properties of CNTs allow them to be used in field emission sources, scanning probes, and nanodevices. In addition to NTs, other 1D nanostructures, such as NWs and NRs, have also been studied. There is no clear standard for distinguishing between NWs and NRs. The structure of NWs is different from that of NTs, the difference lies in the cross‐section. The NW cross‐section is cylindrical and solid, while the NT cross‐section is tubular and hollow.

It expresses that one of the dimensions will be on nanoscale, and the remaining two dimensions will be outside of it. The nanofilms, nanoflakes, and nanosheets are examples. Due to their special physical and chemical properties, 2D NMs have been successfully applied in many aspects of technology and have become a key class of materials in condensed matter physics, materials science, and chemistry. The discovery of graphene in 2004 led to numerous exciting breakthroughs in physics, chemistry, nanotechnology, and even biological sciences. There was no doubt that graphene had brought the 2D NM family into the spotlight for its unique electronic, mechanical, and optical properties. Over the past two decades, researchers have witnessed tremendous progress in graphene research. It changes the landscape of many fields of science and technology, especially condensed matter physics. In addition, transition metal disulfide compounds especially those with an atomic thickness (such as transition metal dichalcogenides (TMDs; e.g., MoS_2_, WS_2_, TiS_2_, TaS_2_, MoSe_2_, WSe_2_, MoWS_2,_ etc.)) have emerged as a new class of promising NMs for fundamental research and applications due to their fascinating properties. Moreover, some transition metal oxides (such as MnO_2_ and TiO_2_), and layered double hydroxides (LDHs, such as NiFe‐LDH, NiMn‐LDH, NiAl‐LDH, etc.) can be used in sensors, electrochemical catalysts, and battery electrodes. Recently, many new types of ultrathin 2D NMs, for example, hexagonal boron nitride (h‐BN), polymers, metal–organic frameworks (MOFs), covalent organic frameworks (COFs), and MXenes, have also been explored and further greatly enriched the family of 2D NMs.^[^ ^10^ ^]^ Driven by their extraordinary properties, numerous methods for synthesizing 2D NMs have been developed, for example, mechanical cleavage, ion‐intercalation and exfoliation, liquid exfoliation, CVD, wet‐chemical syntheses, etc. This novel class of NMs has brought many unique features and thus is being probed for numerous promising applications. Notably, the 0D, 1D, and 2D NMs are often referred to as low‐dimensional NMs.

It usually is larger than nanoscale (1–100 nm) dimensions in three dimensions, commonly referred to as bulk NMs. The 0D, 1D, and 2D NMs are the building blocks of 3D NMs. Moreover, according to the components, NMs can be divided into metal NMs, metal composite NMs, and nonmetallic NMs. Based on the specific structures and compositions, NMs can be applied in many frontier fields, such as energy catalysis, since their physical or chemical properties could be regulated flexibly.

### 2.1 Size Effect for NMs

The size effect of NMs on catalytic activity has been extensively studied. The influence of size on performance is mainly due to the following reasons: 1) size reduction can improve the specific surface area and atomic utilization of electrocatalysts; 2) the electronic structure, surface structure, and metal–support interaction of the catalyst changed significantly with the decrease of particle size; 3) differences in nanoparticle size can affect the generation of different reaction intermediates and thus the selectivity of the final product; and 4) reducing the size of the catalysts can also increase the number of unsaturated coordinating atoms, thereby increasing the activity.^[^ ^11^ ^]^ Based on the unique size effect in NMs, it shows superior catalytic performance in the field of catalysis.^[^ ^12^ ^]^

#### 2.1.1 As the Field of Thermocatalysis

For example, Lu et al. revealed the modulation rule of size effect for the Pd nanoparticles (NPs) in Pd/Al_2_O_3_ catalysts on the catalytic reaction with the change of size.^[12a]^ High activity and selectivity were achieved in the Pd‐catalyzed selective oxidation of benzyl alcohol to benzaldehyde with important application background. The specific results were described in the following. In the process of studying the size effect of metal Pd catalysts in this reaction, it was found that the catalytic activity and selectivity of Pd particles showed a “Volcanic” trend with particle size. At the large size of 19.1 nm, although the selectivity was 93% higher, the activity was poor (turnover frequencies (TOFs) < 1 × 10^4^ h^−1^). For 4.2 nm samples, although the activity was higher (TOFs = 4.3 × 10^4^ h^−1^), the selectivity was lower (72%). The selectivity was 87% higher at 2.1 nm, but the activity was poor (TOFs < 1.7 × 10^4^ h^−1^). In situ X‐ray photoelectron spectroscopy (XPS) measurements demonstrated that the Pd 3 d binding energy gradually shifted to higher positions with an amplitude of 1.3 eV as the Pd particle size decreased. The Pd 3d binding energy XPS increased dramatically below 4.2 nm, indicating a significant change in the corresponding size structure. The size effect significantly changed the reaction pathway of hydrogen removal and altered the corresponding catalytic activity and selectivity.

#### 2.1.2 As the Field of Electrocatalysis

**Figure 2:**
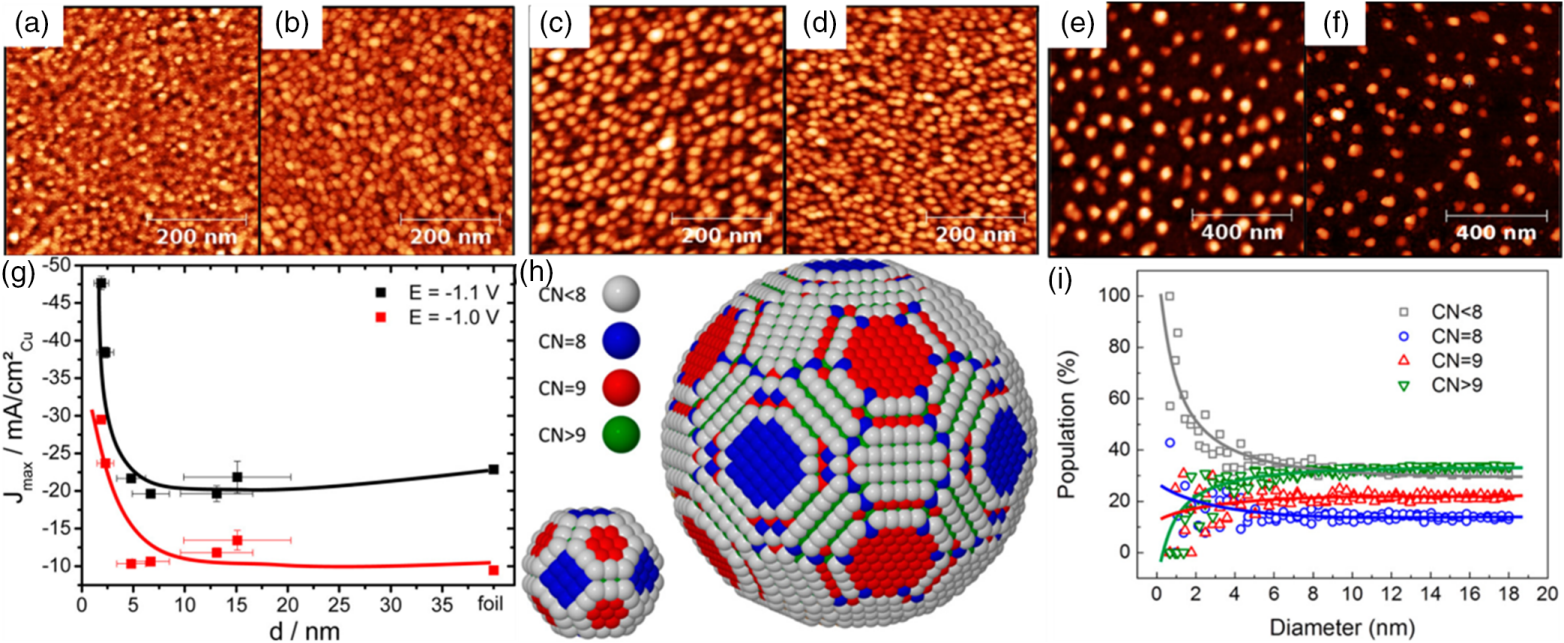
a–f) Tapping‐mode atomic force microscopy images of micellar Cu NPs: (a) S1, (b) S2, (c) S3, (d) S4, (e) S5, and (f) S6. g) Particle size effect during catalytic CO_2_ electroreduction. h) Ball models of spherical Cu NPs with 2.2 and 6.9 nm diameters. Surface atoms are color‐coded according to their first neighbor coordination number (CN), CN < 8 (gray), CN = 8 (blue), CN = 9 (red), CN > 9 (green). i) Population of surface atoms with a specific CN as a function of particle diameter. a–i) Reproduced with permission.^[12b]^ Copyright 2014, American Chemical Society.

In 2014, Strasser et al. investigated the particle size effect in the electroreduction of CO_2_ catalyzed by size‐controlled copper NPs.^[12b]^ Six Cu NPs catalysts with different average sizes between 1.5 and 15.1 nm were obtained by micellar‐based nanoparticle preparation method (**Figure** **2**a–f). At two different electrode potentials (*E* = −1.1 and −1.0 V reversible hydrogen electrode (RHE)^−1^), the dependence of the CO_2_ reduction activity of the catalyst on particle size was investigated, and the results showed that the nanoscale spherical copper surface had a significant activity enhancing size effect (Figure 2g). By simulating the surface atomic coordination numbers of spherical Cu NPs (Figure 2h,i) and combined with theoretical calculations (density functional theory (DFT)), it is concluded that the change in the number of low‐coordination surface sites and their stronger chemisorption were associated with increased H_2_ and CO selectivity, higher catalytic activity, and lower hydrocarbon selectivity.

### 2.2 Confinement Effect for NMs

From a special perspective, the confinement effect in catalysis is “through the confinement of a certain physical state (such as nano‐state), the intrinsic properties of the system (such as structure, electronic state, etc.) are changed, thereby regulating the catalytic performance of the system.”^[^ ^13^ ^]^ This unique nanoconfinement effect widely exists in various thermal‐, photo‐, and electrocatalytic research systems, and further promotes the enhancement of catalytic performance.^[^ ^14^ ^]^ Using the interfacial confinement effect on the catalytic structure and electronic properties, the deactivation of the metal in the catalytic active center and the failure of catalytic function that are prone to occur in traditional heterogeneous catalysts in the catalytic process (especially the catalytic oxidation reaction) can be improved. In recent years, many carbon NMs such as graphene and carbon nanotubes have been used as research objects. Furthermore, the 2D or 3D confinement effect of such NMs is used to carry out related research on catalytic reactions. The research results found that the nanoconfinement effect can promote the reduction of the activation barrier of catalysis, thereby improving the catalytic efficiency. The nanoconfinement effect of the supports can also achieve the effect of regulating the catalytic performance of the supported catalyst without changing the composition of the catalyst, thereby improving the catalytic efficiency.

#### 2.2.1 As the Field of Thermocatalysis

**Figure 3:**
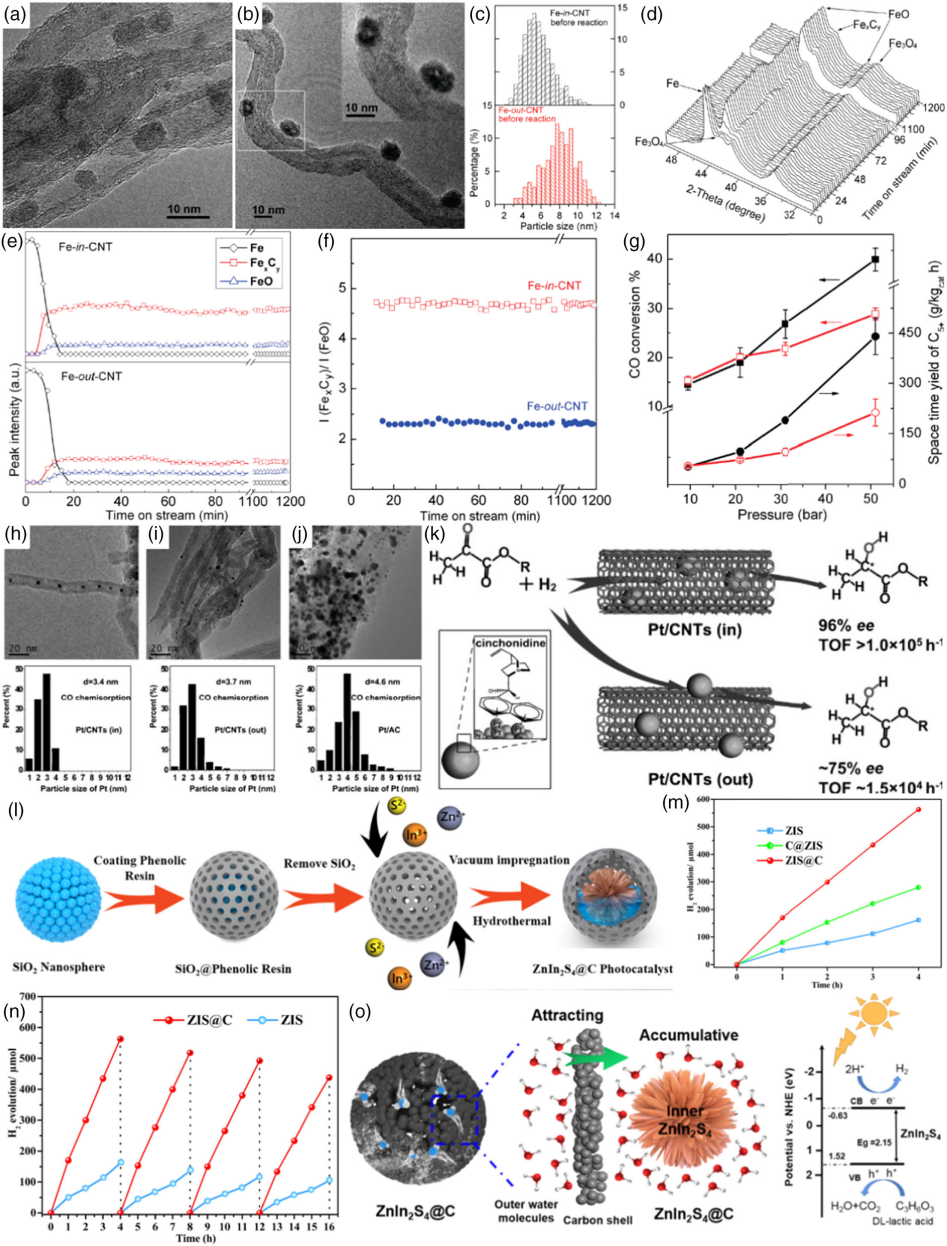
a–c) TEM images (a,b) and particle size distribution (c) of the activated catalysts: a) Fe‐in‐carbon nanotube (CNT) and b) Fe‐out‐CNT before reaction. d–f) Crystal phase evolution of iron catalysts under conditions close to those of the Fischer–Tropsch synthesis (FTS) reaction during the time on‐stream: diffraction patterns of Fe‐in‐CNT (d); intensity of the diffraction peaks of metallic iron, iron carbide, and oxide (e); relative ratio of the integral peak areas of Fe_*x*_C_*y*_/FeO (f). g) FTS activity of Fe‐in‐CNT and Fe‐out‐CNT at 270 °C as a function of pressure. a–g) Reproduced with permission.^[14a]^ Copyright 2008, American Chemical Society. h–j) Low‐magnification TEM images and the platinum particle‐size distribution for Pt/CNTs (in) (h), Pt/CNTs (out) (i), and Pt/activated carbon (AC) (j). k) Asymmetric hydrogenation of α‐ketoesters on the Pt nanoparticles encapsulated within the CNTs (Pt/CNTs (in)) and adsorbed onto CNTs (Pt/CNTs (out)) with cinchonidine as a chiral modifier. h–k) Reproduced with permission.^[14b]^ Copyright 2011, Wiley‐VCH. l) Schematic illustration of the synthesis plan for ZIS@C. m) Hydrogen evolution performance for as‐prepared samples (λ ≥ 420 nm). n) Cycling test for ZIS and ZIS@C samples (λ ≥ 420 nm). o) Schematic illustration of the process of capturing the water molecules and a potential photocatalytic mechanism for hydrogen evolution reaction (HER). l–o) Reproduced with permission.^[14e]^ Copyright 2021, American Chemical Society.

For example, Bao et al. investigated the confinement effects of iron catalysts anchored on CNTs on the catalytic activity in Fischer–Tropsch synthesis (FTS).^[14a]^ TEM characterization showed that the iron oxide particles in Fe_2_O_3_‐in‐CNT and Fe_2_O_3_‐out‐CNT catalysts were very similar in size. Even after 5 h of activation at 350 °C in pure H_2_, the encapsulated Fe particles only grow slightly, and about 80% of the particles fell in the 4–8 nm range. The Fe‐out‐CNT grew to 6–10 nm, which was not significantly larger than the former (**Figure** **3**a–c). The reducibility of iron oxides was explored by H_2_ temperature‐programmed reduction (TPR) and CO‐TPR monitored by in situ XRD (Figure 3d). It was concluded that the phase transition of Fe_2_O_3_‐in‐CNT took place at much lower temperatures than Fe_2_O_3_‐out‐CNT at each reduction step. Combined with TEM, the authors suggest that particle size differences may not be the key reason for the markedly altered iron oxide reducibility observed in H_2_‐TPR and CO‐TPR. After in situ activation, the Fe_*x*_C_*y*_/FeO ratio of Fe‐in‐CNT was 4.7 after 15 min of reaction at 270 °C and 9.5 bar, and remained unchanged for 20 h. In contrast, this ratio in Fe‐out‐CNT was ≈2.4, which was much lower than that in the previous catalyst (Figure 3e,f). This resulted in twice the yield of C_5+_ hydrocarbons on the encapsulated iron catalysts compared to the iron catalysts outside the CNTs and more than 6 times that of the activated carbon supported iron catalysts. With the increase in pressure, the yield of C_5+_ hydrocarbons was obviously improved (Figure 3g). The improvement of catalytic activity was attributed to the role of iron catalyst confined within the CNT channels via the confinement effect.

In 2011, Li et al. reported three different Pt‐based catalysts (Pt/CNTs (in), Pt/CNTs (out), and Pt/activated carbon (AC) modified with cinchonidine (CD) for the asymmetric hydrogenation of α‐ketoesters.^[14b]^ The TEM images showed (Figure 3h–j) that most of the platinum nanoparticles of Pt/CNTs (in) were uniformly distributed inside the CNT nanochannels, and the Pt nanoparticles of Pt/CNTs (out) were dispersed on the outer surface of the CNTs. The results showed that the activity and enantioselectivity of Pt/CNTs (in) nanocatalyst confined to CNT nanochannels significantly increased the asymmetric hydrogenation of α‐ketoesters using CD as chiral modifiers, when the CD‐modified Pt catalysts were encapsulated in CNTs (Figure 3k). The authors believed that the easily enriched CNT nanochannels for CD and reactants were the main reasons for the high activity and enantioselectivity of the catalyst through the nanoconfinement effect.

#### 2.2.2 As the Field of Photocatalysis

In 2021, Yang et al. successfully synthesized a nanoconfined ZnIn_2_S_4_@C photocatalyst by facile encapsulating ultrathin ZnIn_2_S_4_ (ZIS) into the nanoconfined cavity of microporous carbon nanocage (MCN) by vacuum impregnation and selective etching (Figure 3l).^[14e]^ The authors used ZIS@C, C@ZIS (ZIS coated on the outside of the carbon shell), and ZIS (solid carbon spheres) for the hydrogen evolution reaction (HER). The results demonstrated that the ZIS@C photocatalyst exhibited a superior HER activity of 564 μmol over a 4 h reaction time, which was 3.5 times that of pristine ZIS of 282 μmol (Figure 3m). The long‐term stability test demonstrated that the activities of ZIS and ZIS@C with Pt nanoparticles‐loaded decreased by 30% and 20% over four cycles, respectively (Figure 3n). The authors believed that the reason for the high activity of the ZIS@C catalyst was that the hydroxyl functional groups on the outer carbon cages can increase the affinity of ZIS@C for water, resulting in the aggregation of water molecules around the outer carbon shell. Then, water molecules can be adsorbed inside the nanocavity through the nanoconfined effect (Figure 3o).

### 2.3 Defect Effect for NMs

Defects are ubiquitous in nanocatalysts, and the contribution of defects to catalytic activity has long been overlooked due to the lack of direct evidence. Fortunately, with the help of fine characterization techniques and theoretical calculations, researchers are increasingly aware of the importance of defects, and even believe that defects are directly involved in electrocatalytic reactions.^[^ ^15^ ^]^ Defect engineering to tune the surface/interface electronic structure of electrocatalysts, optimize the adsorption energy of intermediate products, and improve the catalytic performance of catalysts has attracted more and more attention.^[^ ^16^ ^]^ Electrocatalysis is one of the most promising applications for NMs in the HER, oxygen evolution reaction (OER), and CO_2_ reduction.^[^ ^17^ ^]^

#### 2.3.1 As the Field of Electrocatalysis

**Figure 4:**
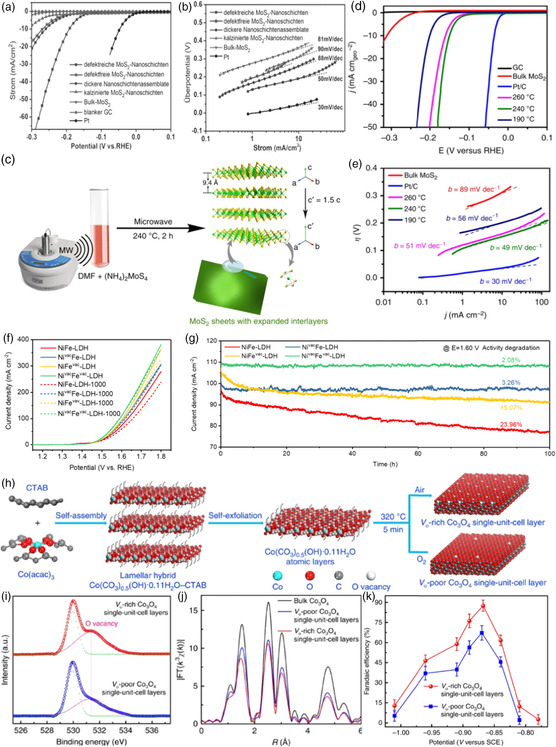
a) Polarization curves of various samples and b) the corresponding Tafel plots. a,b) Reproduced with permission.^[^ ^18^ ^]^ Copyright 2013, Wiley‐VCH. c) Schematic illustration of the synthesis of ET&IE MoS_2_. Green and yellow balls indicate Mo and S atoms, respectively. d) Polarization curves for HER on bare glassy carbon electrode and modified glassy carbon electrodes comprising MoS_2_ nanosheets synthesized from different temperatures, bulk MoS_2_, and a high‐quality commercial Pt/C catalyst. e) The Tafel plots for the various catalysts derived from (d). c–e) Reproduced with permission.^[^ ^20^ ^]^ Copyright 2015, The Authors, published by Springer Nature. f) Electrochemically active area (ECSA)‐normalized polarization curves for NiFe‐layered double hydroxide (LDH), Ni^vac^Fe‐LDH, NiFe^vac^‐LDH, Ni^vac^Fe^vac^‐LDH before and after 1000 cyclic voltammetries (CVs). g) Chronoamperometry test at 1.6 V versus reversible hydrogen electrode (RHE) for the four LDH samples. f,g) Reproduced with permission.^[^ ^21^ ^]^ Copyright 2021, Wiley‐VCH. h) Scheme for the formation of V_o_‐rich and V_o_‐poor Co_3_O_4_ single‐unit‐cell layer, respectively. i) O 1s XPS spectra of V_o_‐rich and V_o_‐poor Co_3_O_4_ single‐unit‐cell layers. j) The corresponding Fourier transforms FT(*k* ^*3*^ *χ*(*k*)) of Co K‐edge extended X‐ray absorption fine structure (XAFS). k) Faradaic efficiencies of formate at different applied potentials. h–k) Reproduced under the terms of the CC‐BY Creative Commons Attribution 4.0 International license (https://creativecommons.org/licenses/by/4.0).^[^ ^22^ ^]^ Copyright 2017, The Authors, published by Springer Nature.

Compared with traditional noble metal catalysts such as Pt NPs, MoS_2_ nanosheets had the advantages of low cost, high chemical stability, and good catalytic performance. However, pure MoS_2_ had problems such as limited active sites and inherent poor electrical transport properties. Therefore, scientists have developed different strategies to improve the catalytic performance of MoS_2_ in the HER. Xie et al. proposed a novel method for the controllable fabrication of defect‐rich MoS_2_ ultrathin nanosheets.^[^ ^18^ ^]^ Additional active edge sites were exposed due to a large number of defects present in the ultrathin nanosheets that partially rupture the inert basal plane. In turn, the defect‐rich MoS_2_ ultrathin nanosheets exhibited higher HER activity than the amorphous MoS_2_ nanosheets. The MoS_2_ ultrathin nanosheets had a small onset overpotential of 120 mV (**Figure** **4**a), a large cathodic current density, and a small Tafel slope of 50 mV dec^−1^ (Figure 4b). Jaramillo et al. synthesized mesoporous MoS_2_ nanosheets with a highly ordered double helix structure.^[^ ^19^ ^]^ In the experiment, the author employed the electrodeposition of the Mo source into a silica template to obtain double‐gyroid MoS_2_ materials via regulating various deposition times to control the film thickness, followed by sulfidization with H_2_S. Furthermore, the double‐gyroid MoS_2_ showed superior activity for the HER with a Tafel slope of ≈50 mV per decade. The high surface curvature of this mesoporous‐structured catalyst exposes most of the edge sites, leading to excellent activity for electrocatalytic hydrogen evolution.

Sun et al. synthesized MoS_2_ nanosheets with edge‐to‐edge and interlayer extension properties by means of a microwave‐assisted strategy (Figure 4c).^[^ ^20^ ^]^ The nanosheets exhibited high kinetic indices with an onset potential of −103 mV (Figure 4d), a Tafel slope of 49 mV per decade (Figure 4e), and an exchange current density of 9.62 × 10^−3^ mA cm^−2^. In addition, Waterhouse et al. successfully prepared the 2D NiFe LDH, then by introducing cation‐vacancies into the NiFe LDH basal plane to achieve both high OER activity and stability.^[^ ^21^ ^]^ The OER activity of the obtained samples showed the following order: Ni^vac^Fe^vac^‐LDH > NiFe^vac^‐LDH > Ni^vac^Fe‐LDH > NiFe‐LDH (Figure 4f), suggesting the greatly improved OER performance through introducing M^3+^ site vacancies. Furthermore, the cation vacancy engineering improved the OER long‐term stability of NiFe‐LDH (Figure 4g). Xie and co‐workers successfully fabricated V_o_‐rich and V_o_‐poor Co_3_O_4_ single‐unit‐cell layers via a lamellar inorganic–organic hybrid intermediate strategy and further explored the carbon dioxide reduction reaction (CO_2_RR) activity (Figure 4h).^[^ ^22^ ^]^ The O 1s core level of XPS spectra demonstrated the existence of an oxygen vacancy (Figure 4i). Furthermore, the Co K‐edge extended X‐ray absorption fine structure (EXAFS) data revealed their distinct local atomic in V_o_‐rich Co_3_O_4_ single‐unit‐cell layers (Figure 4j). The V_o_‐rich Co_3_O_4_ electrodes achieved 87.6% Faradaic efficiencies (FEs) for formate production at a moderately negative potential of −0.87 V vs SCE (Figure 4k).

### 2.4 Strain Effect for NMs

**Figure 5:**
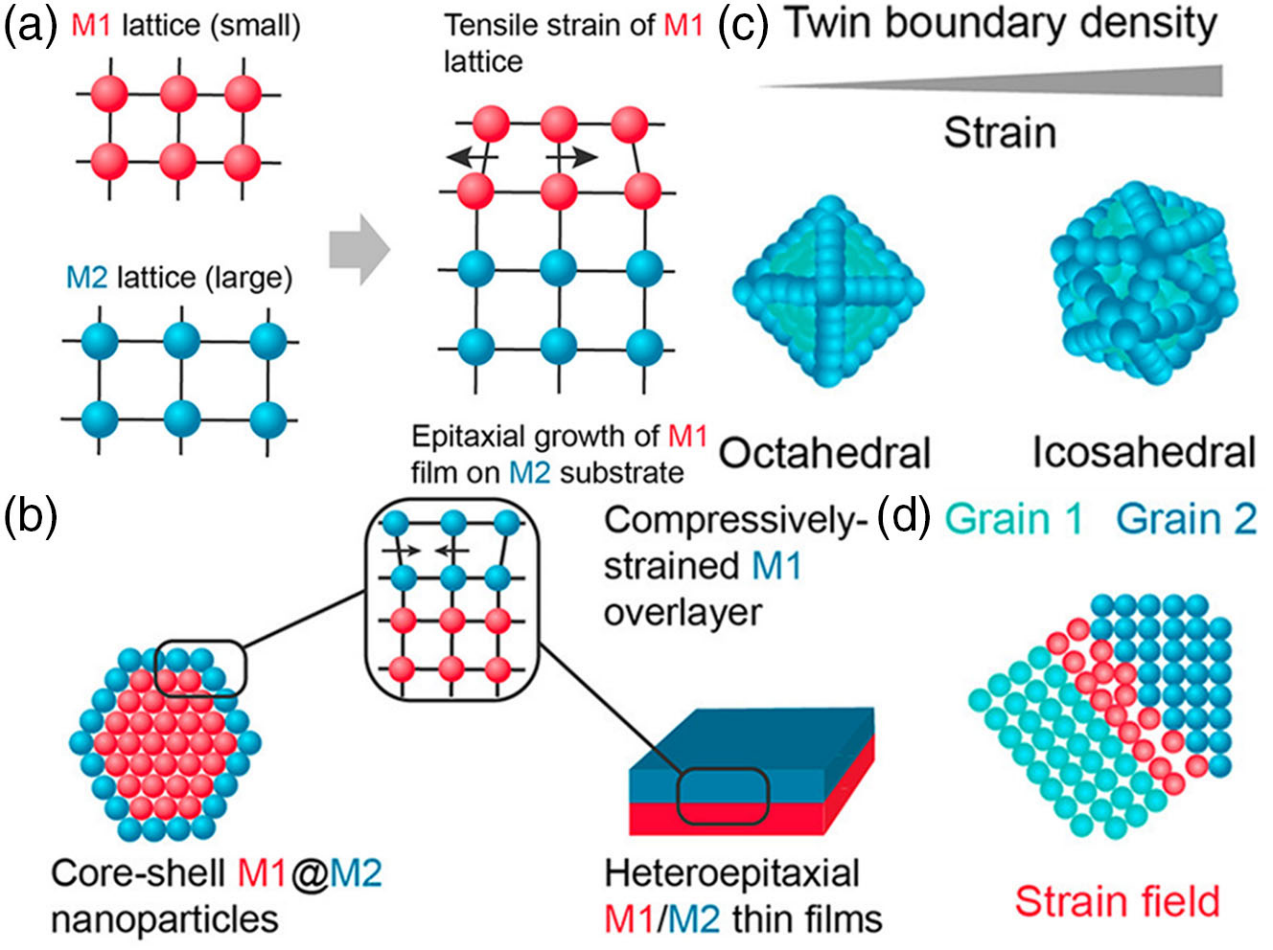
Elastic strain in nanomaterials. a,b) Illustration of lattice‐mismatched epitaxy leading to tensile (a) or compressive (b) strain in bimetallic thin films and nanoparticles. c) Certain nanoparticle geometries result in lattice strain arising from a higher density of twin boundaries. d) The intersection of grains on polycrystalline metal surfaces causes strain due to differing orientation of the lattice at the grain boundary. a–d) Reproduced with permission.^[^ ^23^ ^]^ Copyright 2019, American Chemical Society.

Strain effect in electrocatalysts can be fabricated through stress induced by lattice mismatches, special shapes, or defects. Lattice mismatch‐induced stress is achieved by changing the metal thin film substrate or by epitaxial growing hetero thin films on the surface of nanoparticles. For epitaxial grown hetero‐multilayer materials, lattice mismatch can also be tuned by changing the nanoparticle or substrate composition (**Figure** **5**a,b).^[^ ^23^ ^]^ In addition, twinning can also be formed in nanocrystals, or the shape strains generated in nanoparticles of different shapes are distributed on the sides and edges of the nanoparticles. Defect strains are mainly distributed near grain boundaries or point defects (Figure 5c,d).^[^ ^23^ ^]^ The strain of the electrocatalyst also has a significant effect on the HER, OER, and oxygen reduction reaction (ORR) activity. Studies have shown that straining by introducing lattice mismatch, alloying, or manipulating NM synthesis methods can significantly change the performance of HER, OER, and ORR.^[^ ^24^ ^]^

#### 2.4.1 As the Field of Electrocatalysis

**Figure 6:**
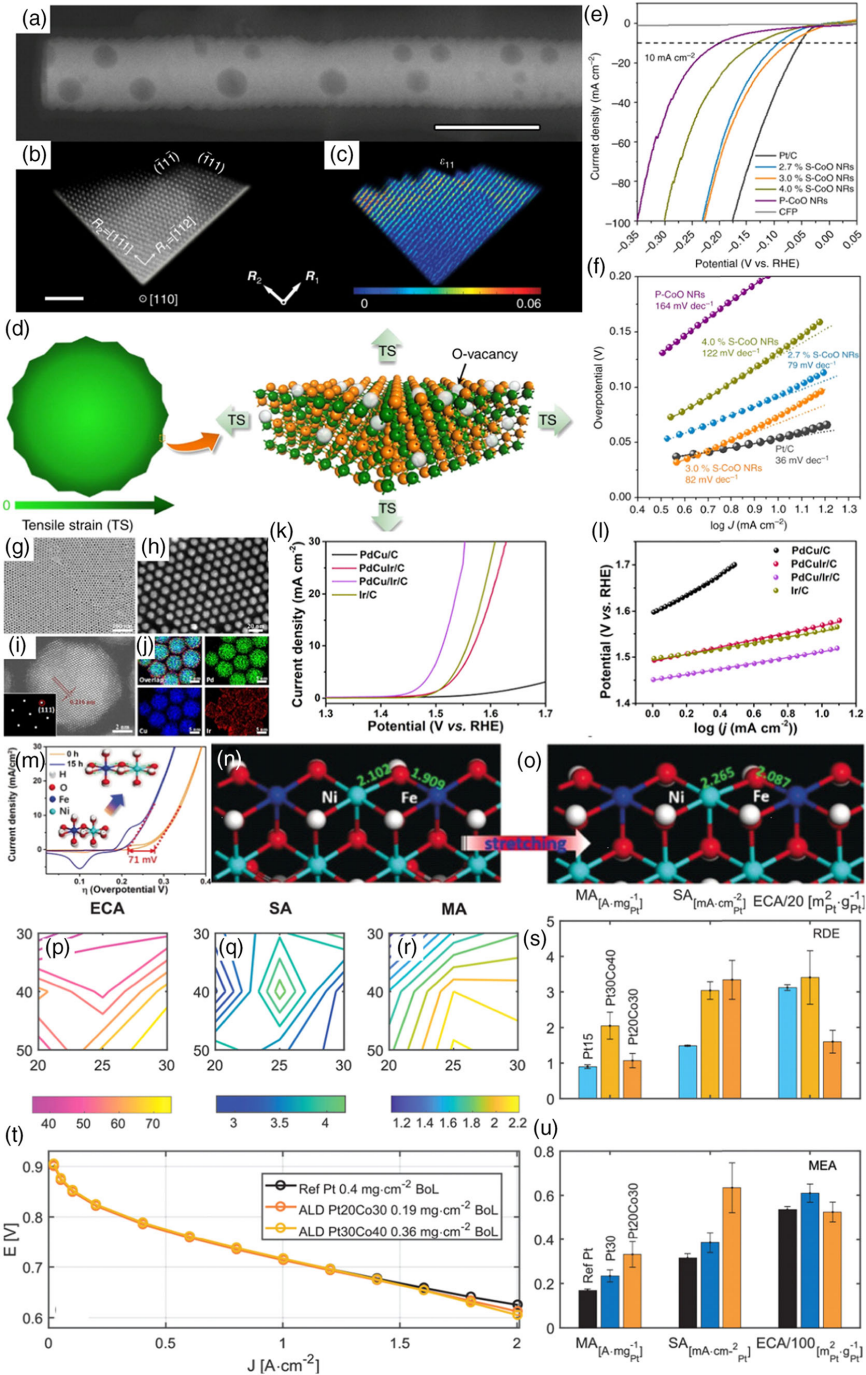
a) Typical HAADF‐STEM image of an individual S‐CoO nanorods (NRs). Scale bar: 100 nm. b) Atomic‐resolution HAADF‐STEM image of two adjacent nano‐sawtooths enclosed with {111} nanofacets, indicating the lattice vectors *R*_1_ and *R*_2_ used as a reference for the strain analysis. Scale bar: 2 nm. c) Contour plots of the strain component *ε*_11_ relative to the reference values. d) Schematic illustration of the formation of abundant O‐vacancies induced by tensile strain on the outermost surface of S‐CoO NRs. e) Linear sweep voltammetry (LSV) of S‐CoO NRs with different tensile strains, P‐CoO NRs, commercial Pt/C catalysts, and carbon fiber paper substrate recorded in 1 m KOH solution with *iR*‐correction. f) Corresponding Tafel plots of the LSV curves in (e). a–f) Reproduced under the terms of the CC‐BY Creative Commons Attribution 4.0 International license (https://creativecommons.org/licenses/by/4.0).^[24c]^ Copyright 2017, The Authors, published by Springer Nature. g) Low‐magnification and h) HAADF‐STEM images, i) atomic‐resolution HAADF‐STEM image (inset is the corresponding fast Fourier transform pattern), j) elemental energy‐dispersive X‐ray spectroscopy mapping of PdCu/Ir core/shell nanocrystals. k) The oxygen evolution reaction (OER) polarization curves. l) The corresponding Tafel slope plots. g–l) Reproduced with permission.^[24d]^ Copyright 2021, Wiley‐VCH. m) CV curves of NiFe‐LDH before and after 15 h of ball‐milling treatment after iR&BET‐correction. n,o) Theoretical calculations of slab model for OER catalysis on NiFe‐LDH with and without ball‐milling treatment. m–o) Reproduced with permission.^[24e]^ Copyright 2018, Wiley‐VCH. p) Electrochemically active area, q) specific activity, and r) mass activity @0.9 V versus RHE of strained Pt/CoO_*x*_ catalysts deposited by varied Pt and CoO_*x*_ ALD cycles. s) Comparison of these performance metrics of Pt30Co40, Pt20Co30, and Pt15 catalysts in the RDE. t) Fuel cell performance of 5 cm^2^ MEAs with Pt20Co30, Pt30Co40, and Pt cathodes. u) Comparison of the performance metrics of the two ALD catalysts versus a reference Pt MEA @0.9 V iR‐free. p–u) Reproduced under the terms of the CC‐BY Creative Commons Attribution 4.0 International license (https://creativecommons.org/licenses/by/4.0).^[24f]^ Copyright 2021, The Authors, published by Wiley‐VCH.

Qiao et al. transformed transition metal oxides (Cobalt (II) oxide NRs, CoO NRs), abundant inactive materials in nature, into highly efficient HER electrocatalysts through strain engineering.^[24c]^ The tensile strain induced by cation exchange was mainly located on the topmost surface of CoO NRs, which was beneficial for fine‐tuning the electronic structure of the surface and thus improving its activity (**Figure** **6**a–c). DFT calculations showed that a tensile strain of 3.0% on CoO {111}‐O surface can reduce the formation energy of O‐vacancies by about 40%, thereby promoting the formation of a large number of O‐vacancies on the surface (Figure 6d). The O‐vacancy acted as the active site of HER to ensure the high activity of S‐CoO NRs. To reveal the relationship between surface strain and HER catalytic activity, the S‐CoO NRs with 2.7%, 3.0% and 4.0% surface strain were synthesized in situ. The results exhibited that polycrystalline CoO NPs (P‐CoO NRs) with low HER activity and large Tafel slope (164 mV dec^−1^) was an inactive HER catalyst; CoO NRs with strained surfaces exhibited enhanced HER activity, decreased Tafel slope, while 3.0% S‐CoO NR exhibited the highest activity (Figure 6e,f).

Moreover, Guo et al. precisely synthesized PdCu/Ir core–shell nanocrystals by controlled deposition of 4 monolayer Ir shells on PdCu nanocrystals and explored the OER.^[24d]^ The TEM and high‐angle annular dark‐field (HAADF)‐STEM mapping of the PdCu/Ir core–shell indicated Ir trace located at the edges while Pd and Cu traces were concentrated at the center (Figure 6g–j). The OER activity of three catalysts showed that PdCu/Ir/C had the lowest onset potential (1.44 V vs. RHE), and the activity order was PdCu/Ir/C < PdCuIr/C≈Ir/C < PdCu/C (Figure 6k). At the current density of 10 mA cm^−2^, the PdCu/Ir nanocrystals displayed a low OER overpotential of 283 mV. The Tafel slope results indicated that the strained PdCu/Ir core–shell can exhibit faster reaction rates and more favorable water oxidation kinetics (Figure 6l). Liu et al. successfully achieved enhanced the bonding strength of NiFe hydroxide with oxidized intermediates by a simple ball milling method.^[24e]^ The prepared NiFe hydroxide with induced tension exhibited excellent oxygen evolution performance. Compared with the untreated sample, the NiFe‐LDH after ball milling for 15 h showed an earlier redox peak (Figure 6m), indicating stronger adsorption to *OH, and earlier OER onset potential (about 1.44 V vs. RHE). To gain theoretical insights into the relationship between lattice strain and OER kinetics, the authors intentionally stretched both the Ni–O and Fe–O bonds by 0.16 Å during DFT calculations for NiFe‐LDH with ball milling (Figure 6n,o). The results showed that the calculated OER overpotential was reduced from 0.55 to 0.48 eV after introducing tensile strain in NiFe‐LDH, and the Gibbs free energy of each elementary step was optimized. In 2021, Xu et al. deposited cobalt oxide and Pt on the atomic layer of carbon supports, and removed the cobalt oxide template in the platinum shell by acid leaching to produce a platinum catalyst with lattice strain.^[24f]^ It was used for the evaluation of catalytic performance under rotating disk electrodes (RDE) and membrane electrode assembly (MEA). The electrochemically active area (ECSA) contour indicated the existence of larger Pt NPs with high platinum and low CoO_*x*_ cycle numbers, while smaller Pt NPs were obtained under high platinum and high CoO_*x*_ cycle numbers conditions (Figure 6p). Furthermore, moderate Pt and CoO_*x*_ cycling can achieve optimal specific activity (Figure 6q). The key mass activity was closely related to high Pt and high CoO_*x*_ cycle numbers (Figure 6r). Due to the improved specific activity, Pt30Co40 (30 Pt and 40 CoO_*x*_ atomic layer deposition (ALD) cycles) were superior to Pt15 (15 Pt ALD cycles), and the mass activity was more than twice that of Pt15 (Figure 6s). Overall, the benefit of specific activity improvement shown in RDE was successfully translated to MEA, but its value was about 5 times lower (Figure 6t,u). The enhanced catalytic performance was mainly attributed to the lattice strain effect of the catalyst.

In conclusion, NMs at the nanoscale exhibited completely different physical and chemical properties, compared to macroscopic bulk materials. Its physical and chemical properties usually showed the following unique phenomena: size effect, defect effect, confinement effect, and strain effect. The above‐mentioned unique phenomenon of nanometer size promoted its catalytic activity as follows: high specific surface area, high electrical conductivity, excellent electron mobility, good mechanical strength, high thermal stability, etc. Based on these unique properties, NMs had widely been used as catalysts or catalyst supports in the field of catalysis and demonstrated excellent catalytic properties of thermocatalysis, photocatalysis, and electrocatalysis.

## 3 Sub‐Nanometer Scale

**Figure 7:**
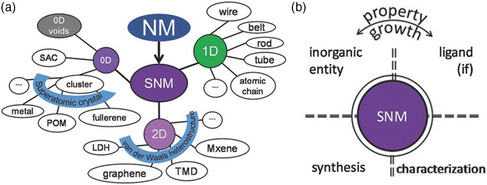
a) The categories of sub‐nanometric materials (SNMs) can be divided into 3 different types based on the material dimensions. b) The foundations of the research on SNMs lies on their synthesis and characterizations, and these two aspects are still obstacles in the studies of SNMs. a,b) Reproduced with permission.^[^ ^27^ ^]^ Copyright 2018, Wiley‐VCH.

With the deepening of nanoscience research, more and more researches have focused on the bottom of the “nanoworld”: a scale of about one nanometer or even less than one nanometer.^[^ ^25^ ^]^ To describe materials at this scale, Wang et al. first coined a pragmatic term “sub‐nanometric material (SNM).”^[^ ^26^ ^]^ The concept of SNM mainly included two aspects: 1) the material size is confined to a few atomic scales in at least one dimension; and 2) it exhibited unique (size‐dependent) properties compared to larger‐sized NMs. Compared with traditional NMs with larger sizes, SNMs display more obvious size effects. It should be noted that, in some cases, the size of SNM is difficult to precisely describe. Therefore, the development of advanced characterization techniques is required to reveal the dimensional changes of materials for rational classification. Usually, drawing lessons from the classification method of nanochemistry, the SNM can also be systematically classified as follows: 1) in the case of 0D SNMs, which exhibit chemical behavior similar to smaller molecules, by adding or subtracting an atom or ligand structure in the host, this will greatly affect and change the physicochemical properties of the materials; 2) for 1D SNMs (usually including wires, ribbons, rods, tubes, chains, etc.), where sub‐nanometer wires are nearly atomically thin in diameter similar to that of polymers, and can be expected to exhibit polymer‐like; and 3) for 2D SNMs, ultrathin materials similar to graphene exhibit unique electronic structures that lead to unexpected properties. The aforementioned classification method is also based on the method of a previously published report, as shown in **Figure** **7**.

### 3.1 Size Effect for Sub‐NMs

**Figure 8:**
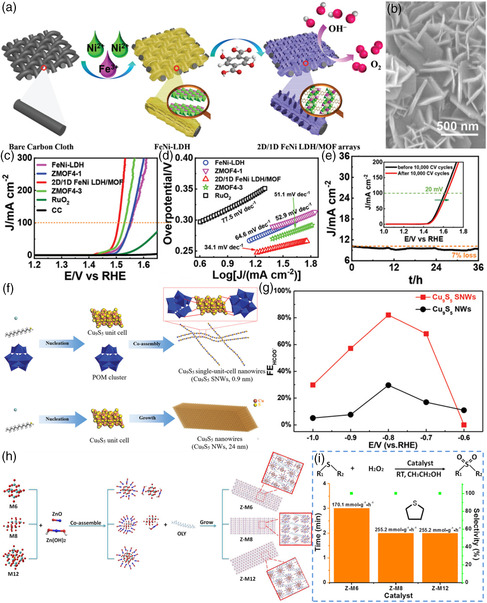
a) Schematic illustration of the preparation of 2D/1D FeNi LDH/ metal–organic frameworks (MOF) arrays. b) SEM images. c) Polarization curves and d) the corresponding Tafel plots of OER on the FeNi‐LDH, ZMOF4‐1, 2D/1D FeNi LDH/MOF, ZMOF4‐3, and RuO_2_ with a 95% *iR* correction and a potential scan rate of 5 mV s^−1^. e) Durability tests of 2D/1D FeNi LDH/MOF in 1.0 M KOH; inset is the polarization curves before and after 10 000 potential cycles without *iR* compensation. a–e) Reproduced with permission.^[28a]^ Copyright 2021, Wiley‐VCH. f) Schematic illustration of preparation of Cu_9_S_5_ SNWs and Cu_9_S_5_ NWs. g) The FE_HCOO_^−^ for Cu_9_S_5_ SNWs (red line) and Cu_9_S_5_ NWs (black line). f,g) Reproduced with permission.^[28f]^ Copyright 2021, Wiley‐VCH. h) Schematic illustration for the formation of 2D HSNSs. i) Oxidation of thioethers catalyzed by three kinds of ZnO–polyoxometalate (POM)‐based 2D HSNSs at 25 °C. The columns chart of catalytic activity for three kinds of catalysts using tetrahydrothiophene (THT) as substrate. h,i) Reproduced with permission.^[28i]^ Copyright 2021, American Chemical Society.

Based on the abovementioned special sub‐nanometer size, they are also confirmed to exhibit good catalytic performance in the field of catalysis.^[^ ^28^ ^]^ In the field of electrocatalysis: For example, Chen et al. reported a sub‐2 nm ultrathin and robust 2D/1D FeNi LDH‐MOF hybrid arrays by in situ strategy (**Figure** **8**a).^[28a]^ The scanning electron microscopy (SEM) image (Figure 8b) demonstrated that the obtained FeNi‐LDHs have well‐defined 2D nanosheet morphology with a very smooth surface and regular arrangement of nanoarrays. Based on unique sub‐nanostructures, the prepared 2D/1D FeNi LDH/MOF sample showed superior electrocatalytic activity with the lowest overpotential of 272 mV under 100 mA cm^−2^ and the lowest Tafel slop of 34.1 mV dec^−1^ (Figure 8c,d), which are significantly better than the control sample, such as FeNi‐LDH, ZMOF4‐1, ZMOF4‐3, and noble metal RuO_2_. Moreover, the 2D/1D FeNi LDH/MOF demonstrated excellent long‐term stability with a negligible loss (only 7% loss) after 36 h of durability test and a negligible positive shift after 10 000 cycles (Figure 8e), further indicating the excellent durability.

Wang et al. successfully fabricated the sub‐1 nm Cu_9_S_5_ NWs (denoted as Cu_9_S_5_ sub nanowires (SNWs)) by introducing polyoxometalate (POM) clusters in the nucleation stage of copper sulfide (Figure 8f), and then explored the electrocatalytic CO_2_ reduction performance.^[28f]^ The Cu_9_S_5_ SNWs exhibited an 82.0% maximum FE_HCOO_^−^ at −0.8 V and superior product selectivity (Figure 8g), compared to Cu_9_S_5_ NWs. Moreover, the Huang group developed a general approach for fabricating subnanometer trimetallic PtNiCo alloy NWs with a diameter of 4 to 5 atomic layer thickness.^[^ ^29^ ^]^ The morphology and structure of subnanometer PtNiCo NWs were further characterized by scanning transition electron microscopy (STEM) and high‐resolution TEM (HRTEM) images. Based on the catalytically active sites on high‐density (111) facets in the subnanometer Pt alloy NWs, the subnanometer Pt alloy NWs demonstrate exceptional ORR catalytic activities.

#### 3.1.1 As the Field of Thermocatalysis

The Wang group successfully exploited the “cluster–nuclei coassembly” method to prepared the superstructures of ZnO–POM‐based 2D hybrid sub‐1 nm nanosheet (HSNS) with introducing ≈1 nm POM clusters into the ZnO/Zn(OH)_2_ system (Figure 8h).^[28i]^ Especially, the Z‐M8 and Z‐M12 catalysts demonstrated the highest mass‐specific activity of 255.2 mmol g^−1^ h^−1^ and 100% selectivity for the oxidation of tetrahydrothiophene (THT) (Figure 8i), indicating excellent catalytic activity and selectivity. Recently, Wang's team reported a new class of graphene‐like 2D SNMs “clusterphene” based on POMs.^[28j]^ The Keggin‐type clusters substituted by rare earth atoms [NdPW_11_O_39_]^4−^ are directly connected by metal–oxygen coordination bonds, and then form a monolayer cluster alkene‐like structure with uniform hexagonal pores under the coating of quaternary ammonium salt cations, and its size can reach several microns. The 2D SNMs exhibit excellent catalytic activity and stability for olefin epoxidation (94% retention in 10 cycles), and their TOF is 76.5 times higher than that of the unassembled moiety.

### 3.2 Polymer‐like Properties for Sub‐NMs

**Figure 9:**
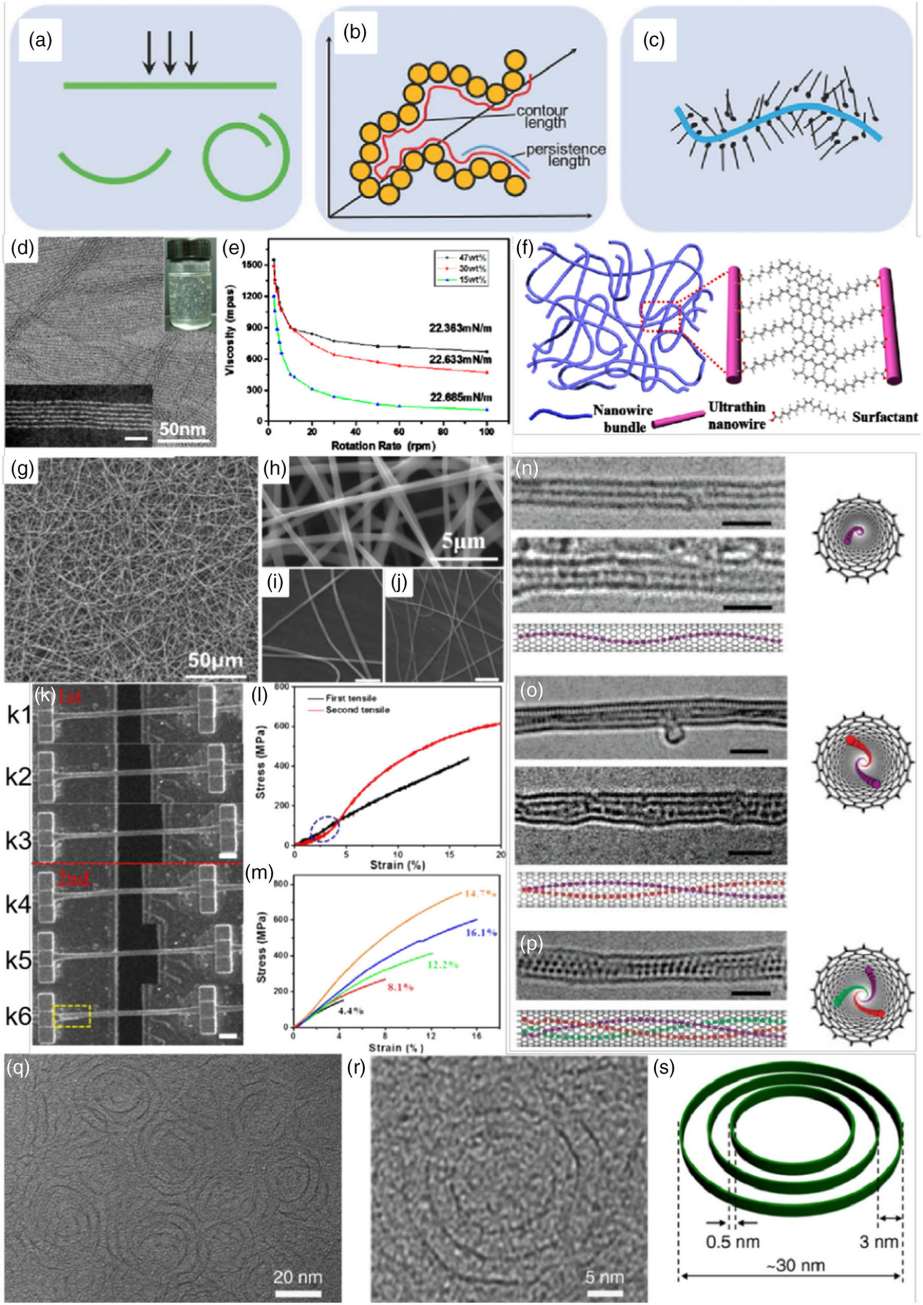
a–c) Three different mechanisms of the origin of the polymer‐like flexibility. a) A simple beam model. b) Conformational entropy. c) The numbers of weak interactions. a,c) Reproduced with permission.^[^ ^27^ ^]^ Copyright 2018, Wiley‐VCH. b) Reproduced with permission.^[^ ^30^ ^]^ Copyright 2013, Wiley‐VCH. d) TEM image showing the sub‐1 nm diameter of the ultrathin NWs for electrospinning. The STEM image (inset bottom left, the scale bar is 10 nm) demonstrates the sub‐1 nm diameter of the ultrathin NWs. The photograph (inset upper right) displays the viscosity characteristic of the NWs dispersion. e) Shear viscosity as a function of the rotation rate of solutions of NWs in octane with different concentrations and the corresponding surface tension. f) Schematic diagram of the entangled network of ultrathin NWs in solution. g–j) Uniform electrospun fibers with superaligned NWs inside. g) SEM images of large‐scale area of electrospinning fibers and h–j) high‐magnification images of the fibers with different diameters of 630 nm (h), 540 nm (i), and 370 nm (j); the scale bar in (c) and (d) is 5 μm. k–m) Loading–unloading tests. k1–3) The first loading cycle with the initial state (k1) and ending strain of 16.8% (k3). k4–6) The second loading cycle until the fracture of the fiber. The scale bar in (k) is 2 μm. l) Corresponding stress–strain curves of the two loading cycles. The blue circle represents the initial tightening process of the second cycle. m) A 5‐time loading–unloading cycle test with gradually increasing strain. d–m) Reproduced with permission.^[^ ^31^ ^]^ Copyright 2017, American Chemical Society. n) A single iodine chain in a thinner single‐walled carbon nanotube (SWCNT) with diameter of 1.05 (0.05 nm and the structure model (side view and top view). o) A double iodine chain in an SWCNT with diameter of 1.30 (0.05 nm and the structure model (side view and top view). p) A triple iodine chain in an SWCNT with diameter of about 1.40 (0.05 nm and the structure model (side view and top view). n–p) Reproduced with permission.^[^ ^32^ ^]^ Copyright 2007, American Chemical Society. q) SEM images of the as‐synthesized mSMO NRs with a yield of 100%; the inset shows a typical image of the free‐standing mSMO NRs, displaying a unique ring‐in‐ring shape. r) HRTEM image of the mSMO NRs. s) The corresponding structural model of the mSMO NRs; the thickness of the ring is about 0.5 nm; the outermost NR size is ≈30 nm; the interval distance between the NRs is ≈ 3.0 nm. q–s) Reproduced under the terms of the CC‐BY Creative Commons Attribution 4.0 International license (https://creativecommons.org/licenses/by/4.0).^[^ ^33^ ^]^ Copyright 2017, The Authors, published by Springer Nature.

Compared with 0D and 2D materials, the 1D SNM has not been fully studied for a long time owing to its high synthesis difficulty and insufficient characterization methods. Fortunately, the 1D SNM was successfully prepared and getting more and more attention, accompanied by the rapid development of advanced characterization techniques of electron microscopy, synchrotron radiation, solid‐state nuclear magnetic resonance, and other technologies. The unique properties of 1D SNM have been experimentally confirmed. The most characteristic properties were the flexibility of the material itself and exhibited polymer‐like properties due to the flexibility. Notably, Wang's research group has made outstanding achievements in the field of SNMs, especially in the 1D sub‐nanometer field. They have carried out research work around NMs with feature sizes approaching 1 nm or even sub‐nanometer scale. Their breakthroughs are mainly based on the following two aspects: 1) when the size of 1D inorganic NMs is limited to about 1 nanometer, and the novel properties of bio‐like macromolecules and polymers will appear; it is possible to open up new avenues for the research and application of inorganic materials by precisely controlling the cluster self‐assembly at the ultrafine nanoscale, and it exhibits excellent properties with both inorganic–organic material properties; and 2) the heterostructures are designed based on the synthesis of ultrafine NMs, and the interface between different materials can be precisely tailored at the sub‐nanoscale or even atomic scale, further carried out the functional regulation such as catalytic properties. Remarkably, their work mainly focuses on the synthesis and properties of 1D sub‐NWs. They put forward three conjectures on the origin of the flexibility of sub‐NWs, namely simple beam model, conformational entropy, and weak intermolecular force, as shown in **Figure** **9**a–c. For the beam model, when a force is applied to the beam, the beam bends accordingly according to its geometric parameters and Young's modulus. In addition, the polymer conformational entropy theory can elucidate the flexibility of SNWs because their morphologies are very similar. For weak intermolecular force, when the size and number of ligands are proportional to the inorganic moiety or even more, they can control the bending behavior. Due to a large number of exposed atoms on the surface, the properties of 1D NMs can be controlled and optimized by surface ligands. It shows great potential in catalysis, energy conversion, etc., but it is still in the early stage of research, and there is still great room for development in this field.

Wang's research group has been exploring the sub‐nanometer field for a long time, and has studied the polymer‐like rheological properties and self‐assembly properties of 1D NWs, as well as many applications of SNMs in the fields of photothermal and electrochemistry. A series of fruitful results have been achieved. In 2017, they prepared sub 1 nm diameter and microscale length GdOOH inorganic NWs superstructures by tuning the viscosity and surface tension of the colloidal NWs solution using electrospinning process methods.^[^ ^31^ ^]^ The TEM image (Figure 9d) and STEM image (bottom left inset image in Figure 9d) demonstrated that the sub‐1 nm GdOOH NWs featured lengths up to the microscale and less than 1 nm in diameters. The inset of Figure 11d exhibited the viscosity caused by the dispersion capturing air bubbles after gentle shaking. When the concentration of NWs was high, the viscosity dramatically increased, and the surface tension decreased slightly (Figure 9e). Furthermore, the flexible dispersed NWs with polymer‐comparable diameter were prone to form bundles and fabricated an entangled NW network accompanied by high concentration and led to viscosity properties (Figure 9f). In addition, the NWs can be directly obtained by electrospinning without adding polymers. Obviously, the SEM image clearly illustrated the morphology of fine‐structured fibers (Figure 9g). The magnification in Figure 9h showed the average diameter of obtained fibers was around 630 nm. In general, the diameters of the NWs could be facilely tuned by changing the experimental parameters, such as the concentration of NWs and tetrabutyl ammonium bromide, and applied voltage (Figure 9g–j). Subsequently, they employed loading–unloading cycle experiments to explore the effect of the fiber NWs orientation on the mechanical property. The SEM images of different tensile points indicated the uniform deformation of fiber during drawing (Figure 9k1–3). The SEM image (Figure 9k4) displayed the state of the tested fiber after the first cycle force was completely removed. The fibers eventually broke, and the broken portion was marked with a yellow box. (Figure 9k6). Figure 9l presented the stress–strain curves of the two tensile cyclings. The experimental results clearly discovered that the sample was first pulled from the bent state in the second tensile test, causing a lower response stress than the first. The hardening response was also explored by a 5‐time loading–unloading cycling test and the added strain in each cycle was controlled at around 4% in Figure 9m. Furthermore, the test demonstrated that the corresponding stress at a point of 4.4% strain increased from 152 to 178, 198, 220, and 305 MPa, respectively. Moreover, Iijima et al. discovered that when the diameter of CNTs was enlarged from about 1.0–1.45 nm, helical chains of di‐iodine atoms and tri‐iodine atoms could be obtained inside CNTs (Figure 9n–p).^[^ ^32^ ^]^ When the diameters of CNTs were greater than 1.45 nm, iodine chains were observed, and only crystallized iodine was formed, inside the CNT or outside. Interestingly, iodine chains were observed when the diameter of CNTs was larger than 1.45 nm, and only iodine crystals were obtained inside or outside the carbon nanotubes. Recently, Wang et al. successfully reported a class of hierarchical sulfur‐doped MoO_2_ nanorings (SMO NRs) materials and it showed the architecture of 0.5 nm wall‐thickness and had a tunable ring‐in‐ring structure.^[^ ^33^ ^]^ The SEM and HRTEM images of the sample with a yield of 100% further confirmed that the as‐prepared SMO NRs were considered a multi‐level structure (Figure 9q,r), and the corresponding structural model was displayed in Figure 9s.

As a bridge connecting atoms/molecules to nanocrystals and even bulk materials, the sub‐nanometer scale represents the limit of the size of materials research. Exploring the basic laws of matter motion at this scale will help improve our synthesis capabilities. Sub‐nanoscale materials have significantly enhanced interactions with external fields, which in turn lead to excellent properties such as catalysis.

## 4 Cluster Scale

Considerable efforts have been made for the synthesis and characterization of traditional nanoparticles. However, a well‐known fact was that no two nanoparticles are the same, which obstructs in‐depth insights into the structure–property relationship of NMs. Clusters were transitional categories between traditional nanoparticles and single atoms, which have countable numbers of atoms, well‐defined arrangement, and limited size range (normally smaller than 2 nm).^[^ ^34^ ^]^ The number of constituent atoms in clusters is only several to several hundreds, which leads to quite different physiochemical properties depending on the number of atoms.^[^ ^35^ ^]^ Unlike the electron energy band structures of bulk metals, the clusters feature discrete electron energies like that of molecules. In a cluster, most of the atoms are directly exposed on the surface or sub‐surface, resulting in much higher atom utilization in catalytic reactions than traditional nanoparticles. In addition, due to the well‐defined structures and limited nuclearity of clusters, the reactant molecules will be absorbed on certain active sites of the clusters, which are convenient for theoretical calculation and leads to high catalytic selectivity.^[^ ^36^ ^]^ With the emergence of these unique and irreplaceable properties, clusters exhibit giant potential for a variety of applications, including catalysis, energy conversion, biology, and optics.^[^ ^37^ ^]^

### 4.1 Size Effect of Clusters

Due to the high specific surface area and the unique physiochemical properties, clusters have attracted increasing research interest in the catalysis field. The size of clusters, the atom arrangement, and the electronic structure of the clusters are three key factors determining their catalytic performance.^[^ ^38^ ^]^ Here, we will discuss the effect of these factors based on typical catalytic reactions, including electrocatalytic (HER, OER,CO_2_RR) and thermal–catalytic (CO oxidation), etc.^[^ ^39^ ^]^

#### 4.1.1 As the Field of Thermocatalysis

**Figure 10:**
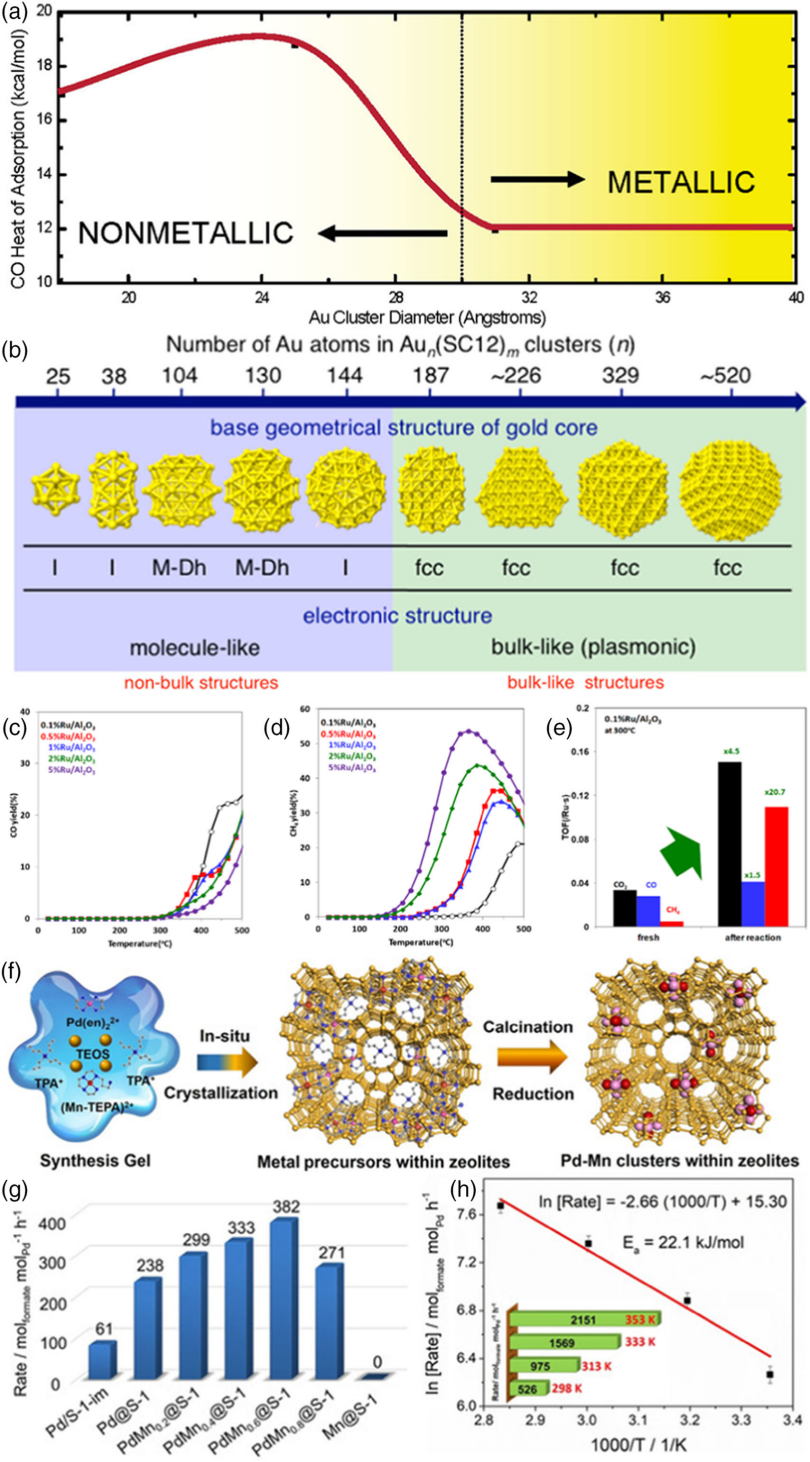
a) The influence of metal cluster size on adsorption energies:  CO adsorbed on Au clusters ranging in size from 1.8 to 3.1 nm anchored on TiO_2_. Reproduced with permission.^[^ ^40^ ^]^ Copyright 2004, American Chemical Society. b) A series of Au_*n*_(SC12)_*m*_ in the *n* range from 38 to ≈520, containing five newly identified or newly isolated clusters. Reproduced with permission.^[38a]^ Copyright 2015, American Chemical Society. c,d) CO and CH_4_ yields on Ru/Al_2_O_3_ catalysts for CO_2_ hydrogenation. e) TOFs for CO_2_ conversion and CO/CH_4_ production over a fresh and re‐activated 0.1% Ru/Al_2_O_3_ catalyst. c–e) Reproduced with permission.^[^ ^41^ ^]^ Copyright 2013, American Chemical Society. f) Schematic of the synthetic procedure of bimetallic PdMn_*x*_@S‐1 catalysts. g) Comparison of the formate generation rates from the CO_2_ hydrogenation over various catalysts. h) CO_2_ hydrogenation over the PdMn_0.6_@S‐1 catalyst in the NaOH solution at different temperatures. Conditions: catalyst (5 mg), 1.5 m aqueous NaOH solution (2 mL), H_2_/CO_2_ (20/20 bar). f–h) Reproduced with permission.^[^ ^42^ ^]^ Copyright 2020, Wiley‐VCH.

In 2004, Goodman et al. demonstrated the structural effects of Au clusters supported on TiO_2_ by the construction of Au clusters ranging in size from 1.8 to 3.1 nm.^[^ ^40^ ^]^ The infrared reflection absorption spectroscopy results revealed that the adsorbed CO vibrational frequency showed blue‐shifts slightly (≈4 cm^−1^) in comparison to bulk Au, which caused the heat of adsorption (−Δ*H*_ads_) to increase sharply from 12.5 to 18.3 kcal mol^−1^ with decreasing cluster size. (**Figure** **10**a). Obviously, the enhanced binding of CO and oxygen for Au clusters with different sizes was vital to revealing the enhanced catalytic activities for TiO_2_ supported on clusters. In 2015, Häkkinen et al. reported a series of Au_*n*_(SC12)_*m*_, such as Au_104_(SC12)_45_, Au_≈226_(SC12)_≈76_, Au_≈253_(SC12)_≈90_, Au_≈356_(SC12)_≈112_, and Au_≈520_(SC12)_≈130_ clusters, in the *n* range from 38 to ≈520 through using reverse‐phase high‐performance liquid chromatography, As can be seen in Figure 10b.^[38a]^ Several experiments of low‐temperature optical absorption spectra, powder XRD and DFT calculations were carried out and unveiled that transition occurs between Au_187_(SC12)_68_ and Au_144_(SC12)_60_ from face‐centered cubic (fcc) to non‐fcc for electronic and geometric.

As the majority of glasshouse air and the most abundant C_1_ resources, CO_2_ sustainable utilization has attracted more and more attention. One acceptable and efficient way is to produce CO, CH_4_, methanol, and other high value‐added products via hydrogenation of CO_2_.^[^ ^43^ ^]^ However, the wide distribution of final products increases the separation cost and restricts the industrial application of this hydrogenation process. By rational controlling the structures of metal clusters, scientists have realized the regulation of selectivity in the hydrogenation of CO_2_. In 2013, Szanyi et al. demonstrated the cluster size dependence of product selectivity in CO_2_ hydrogenation catalyzed by alumina‐supported Ru catalysts.^[^ ^41^ ^]^ The authors prepared a series of Ru/Al_2_O_3_ catalysts with a Ru loading range from 0.1 to 5 wt% and found that the selectivity of CH_4_ increased with the increase of Ru loading, which means the increase in cluster size (Figure 10c,d). This phenomenon is further affirmed by a temperature programmed reaction test on a 0.1% Ru/Al_2_O_3_ catalyst. A dramatic agglomeration of small metal particles (and atoms) into larger clusters was observed and accompanied by an increase in CH_4_ selectivity and a decrease in CO (Figure 10e).

Directly CO_2_ hydrogenation into formic acid or formate has been recognized as a promising way of hydrogen storage. However, developing an efficient heterogeneous catalyst for the industrial application of this process remains challenging. Yan and co‐workers synthesized metallic Pd–Mn clusters encaged within silicalite‐1 zeolites by ligand‐protected method (Figure 10f).^[^ ^42^ ^]^ Notably, the PdMn_0.6_@S‐1 catalysts showed the highest formate generation rate of 382 mol_formate_ mol_Pd_^−1^ h^−1^ at 298 K among all PdMn_*x*_@S‐1 catalysts, which corresponded to a TOF value of 466 h^−1^ (Figure 10g). The generation rate of formate reached 2151 mol_formate_ mol_Pd_^−1^ h^−1^ at 353 K over PdMn_0.6_@S‐1 catalysts, representing the top levels among state‐of‐the‐art heterogeneous catalysts (Figure 10h). The outstanding catalytic performance could be attributed to the formation of ultrasmall bimetallic clusters and the synergic effect of Pd and Mn.

### 4.2 Metal–Support Interactions Effect for Clusters

Compared with NMs, supported nanoclusters not only possessed sufficient metal–metal interactions, but also showed metal–support interactions effect between active species and supports, which are important for enhancing catalytic reactions.^[^ ^44^ ^]^ As an environmental friendly and sustainable hydrogen generation process, HER has aroused increasing interest.^[^ ^45^ ^]^ Pt‐based catalysts are widely used in HER industrial due to their high catalytic activity. Recently, great efforts have been made to explore cheap HER catalysts that could replace Pt.

#### 4.2.1 As the Field of Electrocatalysis

**Figure 11:**
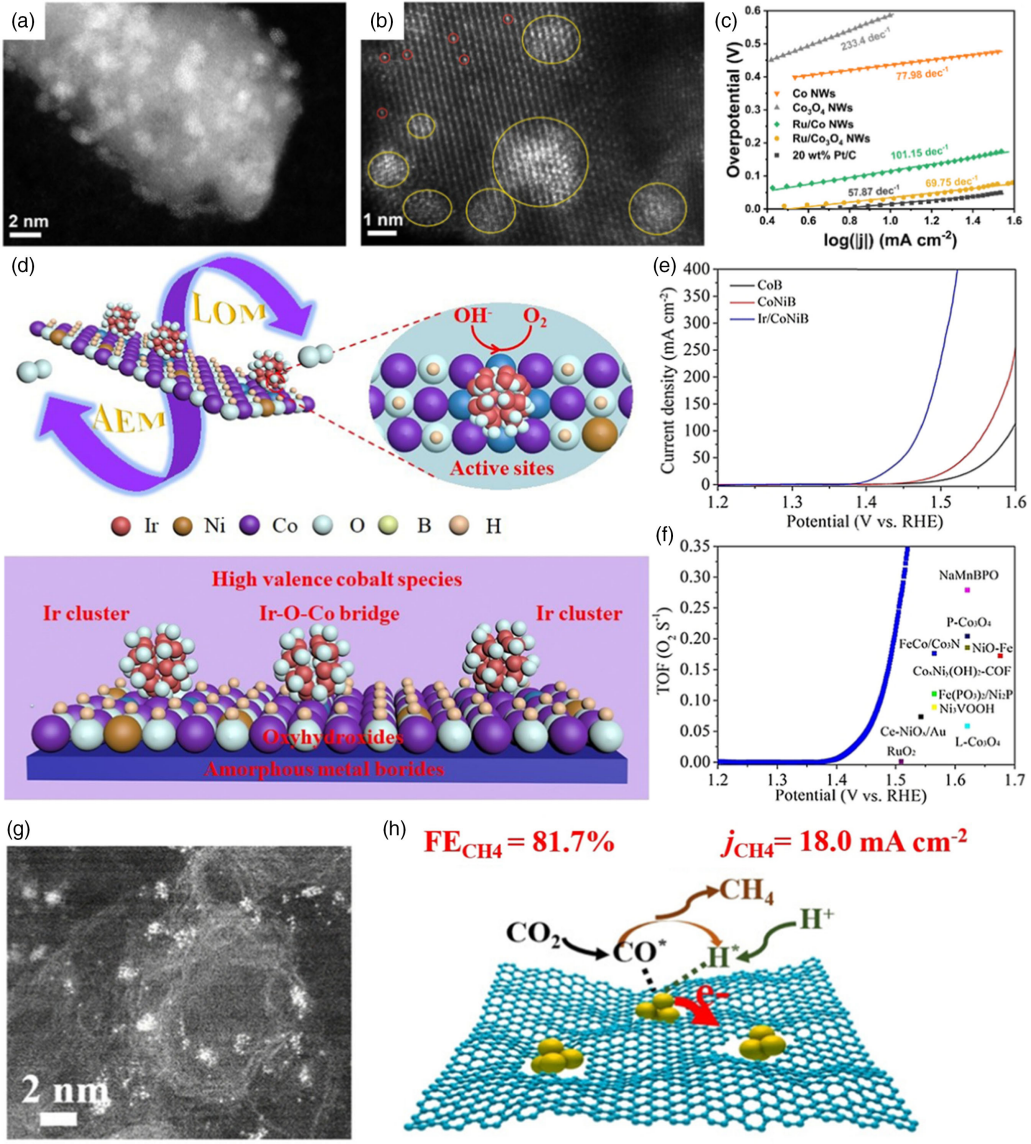
a,b) HAADF‐STEM images of Ru‐clusters/Co_3_O_4_ NWs. c) Tafel plots of Co NWs, Co_3_O_4_ NWs, Ru/Co NWs, Ru/Co_3_O_4_ NWs, and 20 wt% Pt/C for HER. a–c) Reproduced with permission.^[^ ^46^ ^]^ Copyright 2021, Elsevier Ltd. d) Illustration of catalytic mechanism of iridium clusters stabilized surface reconstructed oxyhydroxides on amorphous metal borides. e) OER polarization curves. f) TOF values of Ir‐clusters/CoNiB (blue dots), together with reported catalysts. d–f) Reproduced with permission.^[^ ^48^ ^]^ Copyright 2021, Wiley‐VCH. g) HAADF‐STEM images of Cu clusters/defect‐rich carbon (DRC). h) The carbon dioxide reduction reaction (CO_2_RR) performance and schematic diagram of the mechanism for as‐synthesized Cu clusters/DRC. g,h) Reproduced with permission.^[^ ^49^ ^]^ Copyright 2020, Wiley‐VCH.

Zhou et al. reported the synthesis of Ru clusters loaded on Co_3_O_4_ NWs (Ru/Co_3_O_4_ NWs) and regulation of the bonding environment of Ru nanoclusters by controllable reduction process,^[^ ^46^ ^]^ as shown in **Figure** **11**a,b. Electrochemical test results illustrated that Ru/Co_3_O_4_ NWs possessed an enhanced HER activity and stability than Pt/C catalyst with an overpotential of 31 mV to a current density of 10 mA cm^−2^ (Figure 11c). DFT calculations revealed that the introduction of O species into Ru clusters led to the intrinsic HER activity, which was attributed to excellent metal–support interactions. In addition, Zhao et al. tried to find a promising Cu‐based alloy cluster for HER.^[^ ^47^ ^]^ They developed a DFT‐based high‐throughput screening method, and then optimized and evaluated 7924 candidates of Cu‐based alloy clusters of Cu_55−*n*_M_*n*_ (M = Co, Ni, Ru, and Rh). Generally considering the activity and stability of these catalysts, core–shell CuNi alloy clusters showed a huge potential for HER. Their study proposed a promising way to design and synthesize advanced electrocatalysts for HER. In addition, the clusters also exhibited excellent catalytic performance in terms of OER. For instance, Hou et al. developed efficient iridium clusters catalysts of surface reconstructed oxyhydroxides from amorphous metal borides array to anchor iridium clusters by electrochemical activation strategy (Figure 11d).^[^ ^48^ ^]^ The Ir/CoNiB with the incorporation of Ir clusters presented the best OER activity compared to CoB and CoNiB (Figure 11e), only needing the ultralow overpotentials of 178 mV at 10 mA cm^−2^ and 242 mV at 100 mA cm^−2^, suggesting accelerated OER kinetics in alkaline electrolyte. Moreover, the CoNiB‐supported Ir‐clusters catalysts realized the highest TOF value of 0.37  s^−1^ at the 1.53 V vs. RHE in comparison to the previously reported catalysts (Figure 11f). The results demonstrated that the synergy metal–support interactions effect between iridium clusters and the formation of high valence cobalt oxyhydroxides species supports significantly enhanced water oxidation performance. In addition, the non‐precious metal copper clusters also exhibited good catalytic performance for promoting CO_2_RR to synthesize multi‐carbon fuels. Recently, He et al. used a 600 °C calcination strategy to prepare a Cu cluster catalyst, which is anchored on defect‐rich carbon (DRC) supports.^[^ ^49^ ^]^ Notably, the HAADF‐STEM images demonstrated that the Cu clusters were more prone to locate at the carbon edges positions with more defect sites (Figure 11g). The Cu clusters/DRC achieved a high methane selectivity for CO_2_RR with a maximum FEs of 81.7 % and also exhibited an 18.0 mA cm^−2^ high current (Figure 11h). The synergy of interactions between the Cu clusters and defective carbon further improved the stability and selectivity of the generation of CH_4_ by optimizing the electronic structures of Cu.

So far, the applications of clusters for thermal–catalytic and electrocatalytic reactions have been reviewed. From the aforementioned discussion, it can be seen that the well‐defined atom arrangement of clusters was key factor in determining their catalytic performance. Compared with metal nanoparticles and SACs, fully exposed metal cluster catalysts have unique advantages in catalytic reactions: maintaining 100% atom utilization, and providing abundant surface active sites for catalytic reactions. Therefore, the unique structural advantages of clusters have contributed to their excellent catalytic performance and further are expected to participate in more catalytic conversion processes through structural parameters and coordination state optimization.

## 5 Atomic Scale

Based on the rapid development of advanced characterization techniques, it is now possible to better understand the structure, composition, and function of materials at the atomic level. In this case, the SACs or atomically dispersed catalysts came into being. SACs have attracted much attention in energy and catalysis due to their nearly 100% atom efficiency and unique electronic structure. Compared with traditional nanocatalysts, SACs can maximize the use of each metal atom as an active site, and the active site is uniform. After the concept of single‐atom catalyst was proposed, it received extensive international attention, and single‐atom catalysis has quickly become the research frontier in the field of catalysis. For single‐atom catalysis, the now generally accepted definition: SACs contain only isolated single metal atoms dispersed on supports.^[^ 44, 50 ^]^ The atoms in SACs are not electrically neutral atoms in the physical concept, or positive or negative always has a certain charge; nor are they purely isolated atoms, or strong or weak. Notably, there is always a certain interaction with the support. Without considering the role of the support, it is impossible to talk about SACs. Single atoms emphasized the uniformity of dispersion. Due to the existence of surface heterogeneity of the carrier, that was, the chemical environment of each metal atom was different, and the catalytic activity of each metal single atom may be different.^[^ ^51^ ^]^ It is worth exploring which metal single atoms in the chemical environment played the role of “one to one hundred.”

**Figure 12:**
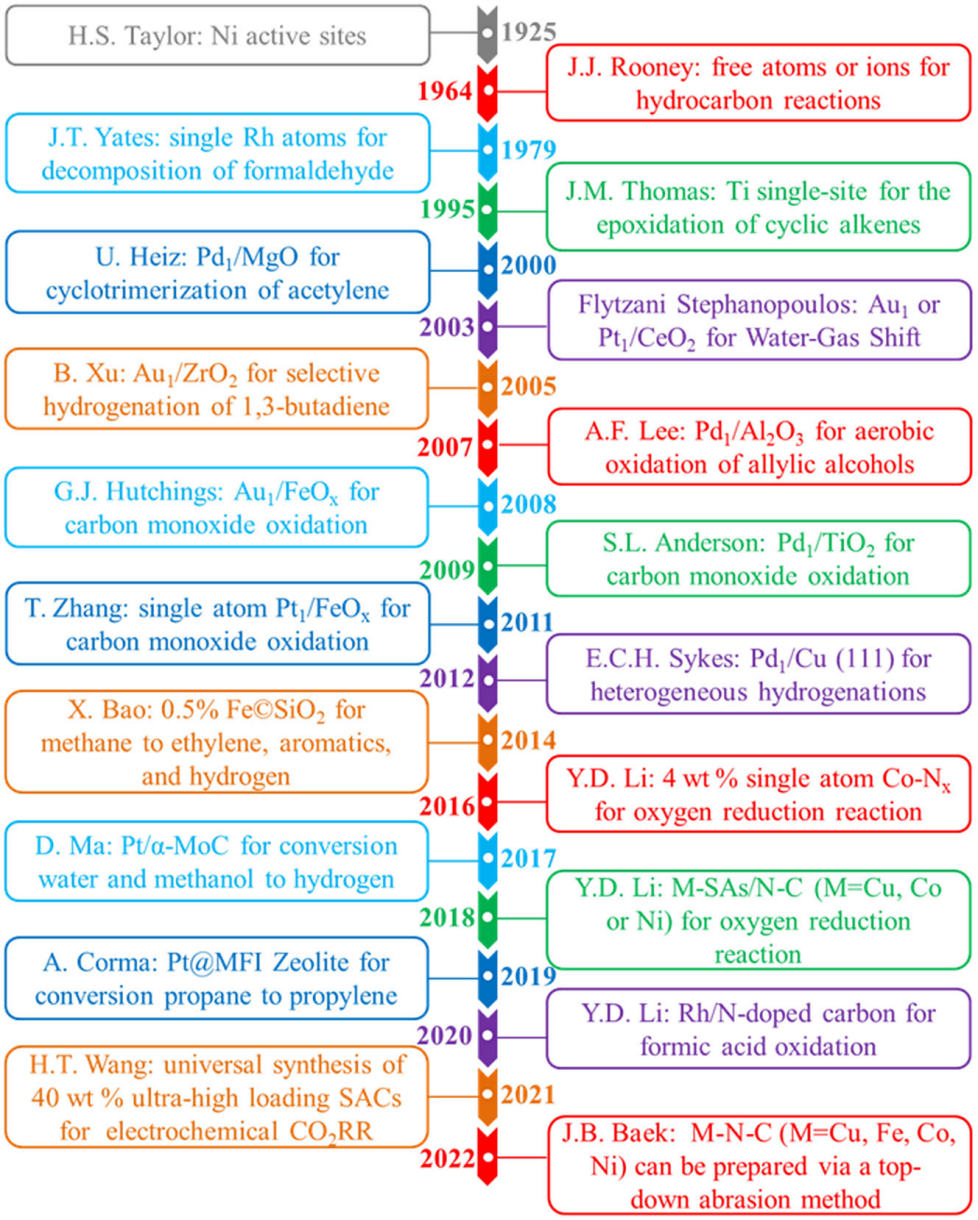
Timeline of the single‐atom catalysts (SACs) with major breakthroughs from 1925 to 2022. Reproduced under the terms of the CC‐BY Creative Commons Attribution 4.0 International license (https://creativecommons.org/licenses/by/4.0).^[^ ^62^ ^]^ Copyright 2022, The Authors, published by Wenzhou University and John Wiley & Sons Australia, Ltd.

In 1925, Taylor et al. early reported that the dispersion state of metal atoms as active sites were mainly isolated ionic or free atoms on the surface of catalysts,^[^ ^52^ ^]^ and this laid the foundation for the subsequent proposal of the single‐atom concept. In 1979, Yates and co‐workers prepared a SAC of single Rh atoms anchored on Al_2_O_3_ supports and probed the isolated rhodium for decomposition of formaldehyde.^[^ ^53^ ^]^ Afterward, Flytzani‐Stephanopoulos successfully fabricated a cationic single‐site Au_1_ or Pt_1_/CeO_2_ catalyst by using a co‐precipitation and leaching strategy in 2003.^[^ ^54^ ^]^ In 2009, Anderson and co‐workers constructed a single Pd atom catalyst of Pd_1_/TiO_2_ and used it to investigate carbon monoxide oxidation catalytic reactions.^[^ ^55^ ^]^ Remarkably, Zhang et al. first reasonable proposed the concept of “SACs” with the help of atomic resolution HAADF‐STEM and XAFS spectra in 2011.^[^ ^7^ ^]^ The proposal of “SACs” concept accelerated its rapid development in various fields in the past ten years. Although SACs played an increasingly significant role on a variety of occasions, there were still challenges in preparing high metal loading mass (usually >1.0 wt%) without aggregation, which severely limited the development of SACs. Fortunately, the Li group fabricated a 4 wt% high metal loading SACs of nitrogen‐doped porous carbon supported single Co atoms with an order‐of‐magnitude significant breakthrough in 2016, which brought infinite possibilities for the practical application of SACs.^[^ ^56^ ^]^ In 2022, Beak et al. fabricated a series of SACs of Fe–N–C, Co–N–C, Ni–N–C, and Cu–N–C via a top‐down abrasion method.^[^ ^57^ ^]^ The synthetic chemicals or solvents (including water) were not employed during the synthesis process and therefore no byproducts or waste were produced. This paved the way for green, universal preparation and wide application of SACs. **Figure** **12** exhibited the timeline of vital breakthroughs in SACs from 1925 to 2022, whose databases were quickly enriched and refined in decades.^[^ 7, 52, 53, 54, 55, 56, 57, 58 ^]^

Recently, the dual/multi‐atom site catalysts also have exhibited excellent catalytic activity in energy conversion and catalysis based on synergistic effects between adjacent metal atoms.^[^ ^59^ ^]^ Compared to SACs, the adjacent metal atoms of dual/multi‐atom site catalysts can also collaborate and have a certain synergistic effect in optimizing the reaction path and promoting efficient conversion between the active sites and intermediates. Diatomic catalysts are extensions of SACs. In diatomic catalysts, dimers of metal atoms are supported on a substrate. The diatomic catalysts currently studied experimentally and theoretically focus on homonuclear metal atom dimers. However, two different metal atoms will form more heteronuclear diatomic catalysts in principle. Therefore, dual atomic or even multi‐atomic catalysts may demonstrate superior activity, higher selectivity, or long‐term stability than SACs. This may contribute to breaking the intrinsic linear scaling relationships between adsorbed intermediate species and reaction energy barriers and further enhance catalytic activity. When it came to atomic‐level materials, it was inevitable to emphasize the supports. Generally, supports were divided into two categories, namely carbon supports and non‐carbon supports. Among, carbon‐based supports, such as graphene, graphdiyne, and porous carbon, have aroused enormous attention and exhibited excellent activity, long‐term stability in the catalytic field.^[^ ^60^ ^]^ Moreover, non‐carbon supported SACs, including metals (alloys), metal oxides, metal hydroxides, metal chalcogenides, metal carbides, metal nitrides, metal phosphides, etc.^[^ ^61^ ^]^

### 5.1 Metal–Support Interactions Effect for Atomic Scale Materials

SACs with unique structures have received great research attention owing to their maximum atom utilization efficiency (nearly 100%), distinct active sites, and high catalytic performance and selectivity, especially for electrocatalysis.^[^ ^63^ ^]^ The noble metal platinum, as a type of platinum group metal (PGM) material, exhibits excellent activity in the field of catalysis, based on its unique electronic structure. Especially, when platinum exists in the form of a single atom with the largest atom utilization efficiency, its catalytic performance will be infinitely expanded. However, its stability becomes a challenge, which was mainly to overcome the agglomeration problem caused by high energy. Thus, it is vital for the construct of efficient and stable Pt‐based SACs to choose appropriate support, which can anchor atomically dispersed species well and inhibit their aggregation and migration. Among the many supports of metal oxides, metal sulfides, alloys, etc., carbon materials have been widely used as the potential support for designing Pt‐based SACs due to their low cost, good conductivity, and rational corrosion resistance. In addition, the electronic structures of carbon supports can be readily regulated by chemical heteroatoms doping methods (i.e., N, P, S, etc.). Recently, through the unremitting efforts of scientific researchers, numerous carbon‐supported platinum SACs (denoted as Pt–N–C) have been successfully reported.^[^ 44, 64 ^]^ Based on metal–support interactions, SACs exhibited excellent catalytic performance.

#### 5.1.1 As the Field of Electrocatalysis

**Figure 13:**
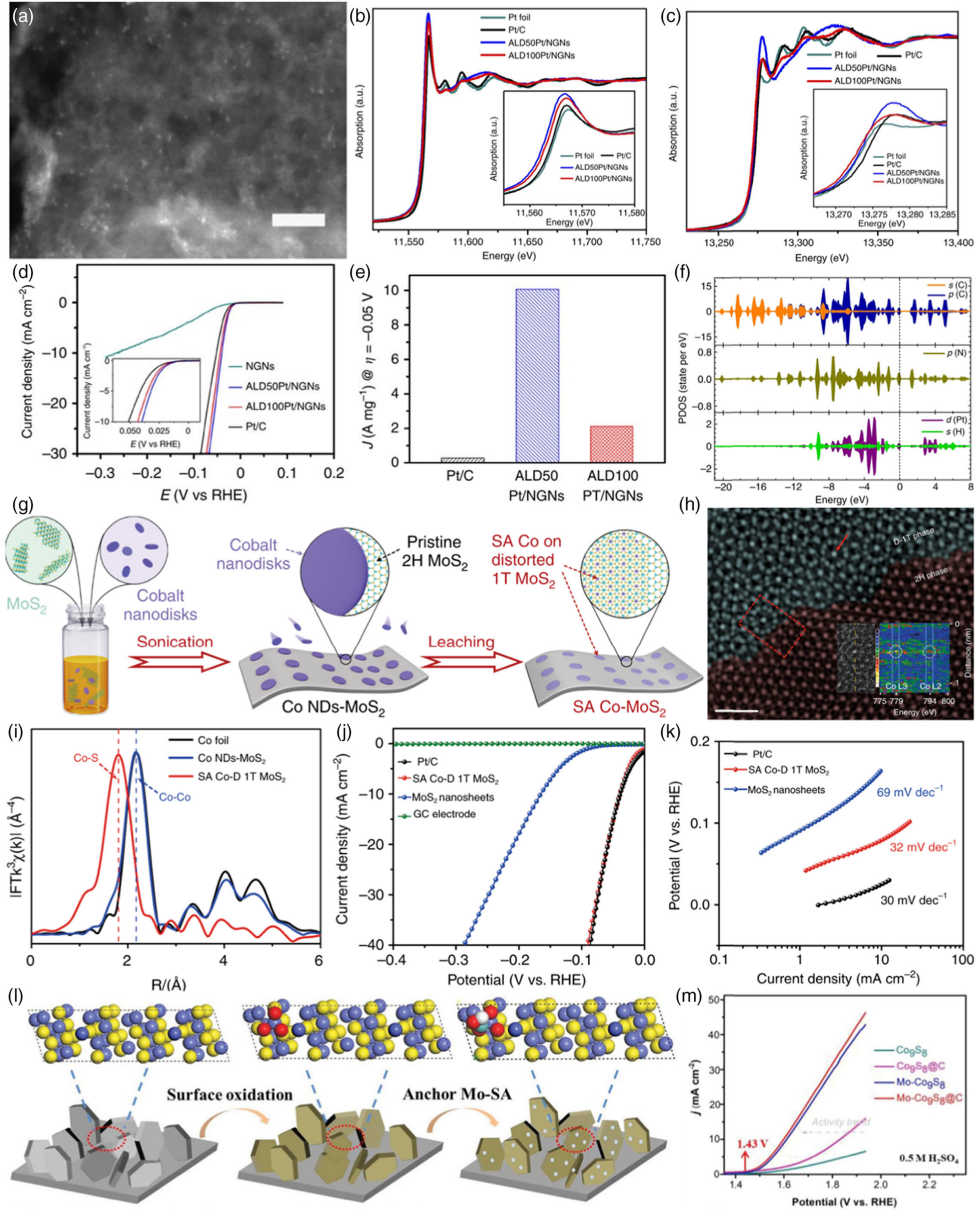
a) ADF STEM images of ALDPt/NGNs samples with 50 ALD cycles. Scale bars: 5 nm. b) Normalized XANES spectra at the Pt L_3_‐edge of the ALDPt/NGNs and references. The inset shows the enlarged spectra. c) Normalized XANES spectra at the Pt L_2_‐edge of ALDPt/NGNs and references. The inset shows the enlarged spectra. d) HER polarization curves for ALDPt/NGNs and Pt/C catalysts. The inset shows the enlarged curves at the onset potential region of the HER. e) Mass activity at 0.05 V (vs. RHE) of the ALDPt/NGNs and the Pt/C catalysts. f) Partial density of states (PDOS) of two H atoms adsorbed on a single Pt atom of ALDPt/NGNs. a–f) Reproduced under the terms of the CC‐BY Creative Commons Attribution 4.0 International license (https://creativecommons.org/licenses/by/4.0).^[64k]^ Copyright 2016, The Authors, published by Springer Nature. g) Schematic diagram of the synthesis process for SA Co‐D 1T MoS_2_. h) Aberration‐corrected HAADF‐STEM image of SA Co‐D 1T MoS_2_, indicating the obvious junction between SA Co‐D 1T MoS_2_ (dark cyan) and pristine 2H MoS_2_ (wine). The inset shows the HRTEM and electron energy loss spectrum of SA Co‐D 1T MoS_2_ (scale bar: 1 nm). i) FT‐EXAFS spectra of SA Co‐D 1T MoS_2_ and bulk cobalt foil at the Co K‐edge. j) Polarization curves of different catalysts tested in Ar‐saturated 0.5 m H_2_SO_4_. k) Tafel plots for the catalysts derived from (j). g–k) Reproduced under the terms of the CC BY Creative Commons Attribution 4.0 International license (https://creativecommons.org/licenses/by/4.0).^[65d]^ Copyright 2019, The Authors, published by Springer Nature. l) Schematic illustration of the synthetic process for Mo‐Co_9_S_8_@C. m) LSV curves for OER of Mo‐Co_9_S_8_@C and the controlled samples in 0.5 m H_2_SO_4_ with a scan rate of 5 mV s^−1^. l,m) Reproduced with permission.^[65m]^ Copyright 2020, Wiley‐VCH.

For instance, Sun et al. fabricated single platinum atoms catalysts with nitrogen‐doped graphene nanosheets (NGNs) supported by atomically dispersed Pt species (namely ALDPt/NGNs) using the ALD technique.^[64k]^ The NGNs were first obtained through post‐heating the graphene in a mixed gas atmosphere of high purity ammonia and argon at a high temperature of 900 °C. Then, the precursors containing platinum of MeCpPtMe_3_ were deposited on the NGNs by ALD with controllable cycles to construct single Pt atoms. The aberration‐corrected annular dark‐field (ADF)‐STEM, and X‐ray absorption near edge spectroscopy (XANES) characterizations indicated the presence of single Pt atoms on NGNs (**Figure** **13**a–c). Impressively, the Pt SACs demonstrated significantly improved HER catalytic activity with a smaller Tafel slope of 29 mV dec^−1^ (Figure 13d,e), compared to the state‐of‐the‐art commercial Pt/C catalysts. Furthermore, the partial density of states (PDOS) results demonstrated that the unique electronic structure was the primary reason for increasing HER activity of the ALD50Pt/NGNs sample (Figure 13f). In addition, although noble metal catalysts have better performance in the energy and catalysis field, researchers have paid more attention to the efficient use of the earth's resource‐rich elements, including iron, cobalt, nickel, manganese, copper, zinc, molybdenum, tungsten, indium, and bismuth elements,^[^ ^65^ ^]^ to reduce costs and improve the promotion and application of energy and catalysis technology. In this section, we summarize recent attempts. For example, Cui and co‐workers developed a single‐atom cobalt (Co) catalyst that Co array was covalently bound onto distorted 1T MoS_2_ nanosheets (denoted as SA Co‐D 1T MoS_2_) via assembly/etching method (Figure 13g).^[65d]^ Furthermore, the aberration‐corrected HAADF‐STEM characterization revealed that single atoms Co (highlighted by red arrow) were homogeneously anchored on the D‐1T MoS_2_ matrix (Figure 13h). Moreover, the EXAFS curve of SA Co‐D 1T MoS_2_ demonstrated that one dominant peak at 1.79 Å was observed and ascribed to Co–S bonds (Figure 13i), and no Co–Co bonds were detected, indicating the presence of atomically dispersed Co species anchored on the supports. The SA Co‐D 1T MoS_2_ presented an extremely small onset overpotential of 42 mV vs. the RHE and low Tafel slope of 32 mV dec^−1^ for HER (Figure 13j,k), which was much better than those of other noble‐metal‐free catalysts and even comparable to that of precious metal 10% Pt/C. Recently, Wang et al. reported a catalyst of single‐atom molybdenum (Mo) that anchored on Co_9_S_8_ support (donated as Mo‐Co_9_S_8_@C) and was used as an efficient and noble‐metal‐free OER (Figure 13l).^[65m]^ Remarkably, the Mo‐Co_9_S_8_@C showed superior OER activity with a lower onset potential at ≈1.43 V (versus RHE) and overpotential of 370 mV at 10.0 mA cm^−2^ (Figure 13m).

### 5.2 Coordination Effect for Atomic Scale Materials

SACs are a very important class of electrocatalysts. Their unique monodisperse structure combines the advantages of homogeneous and heterogeneous catalysts, with maximum metal utilization, excellent catalytic activity, and stability. At the same time, the active site (metal–ligand–substrate, *M*–L_*x*_–C) of SACs is relatively simple to determine and easy to control.^[^ ^66^ ^]^ For example, the catalysts of electronic structure and catalytic performance can be effectively regulated by changing the central metal atom, adjacent coordination elements and coordination numbers. The electronic and geometric structure of the central metal atom can be adjusted by adjusting the coordination environment, thereby changing the absorption activity of reactants or active species to the metal atom site, thereby affecting the catalytic performance.^[^ 51, 67 ^]^

#### 5.2.1 As the Field of Electrocatalysis

**Figure 14:**
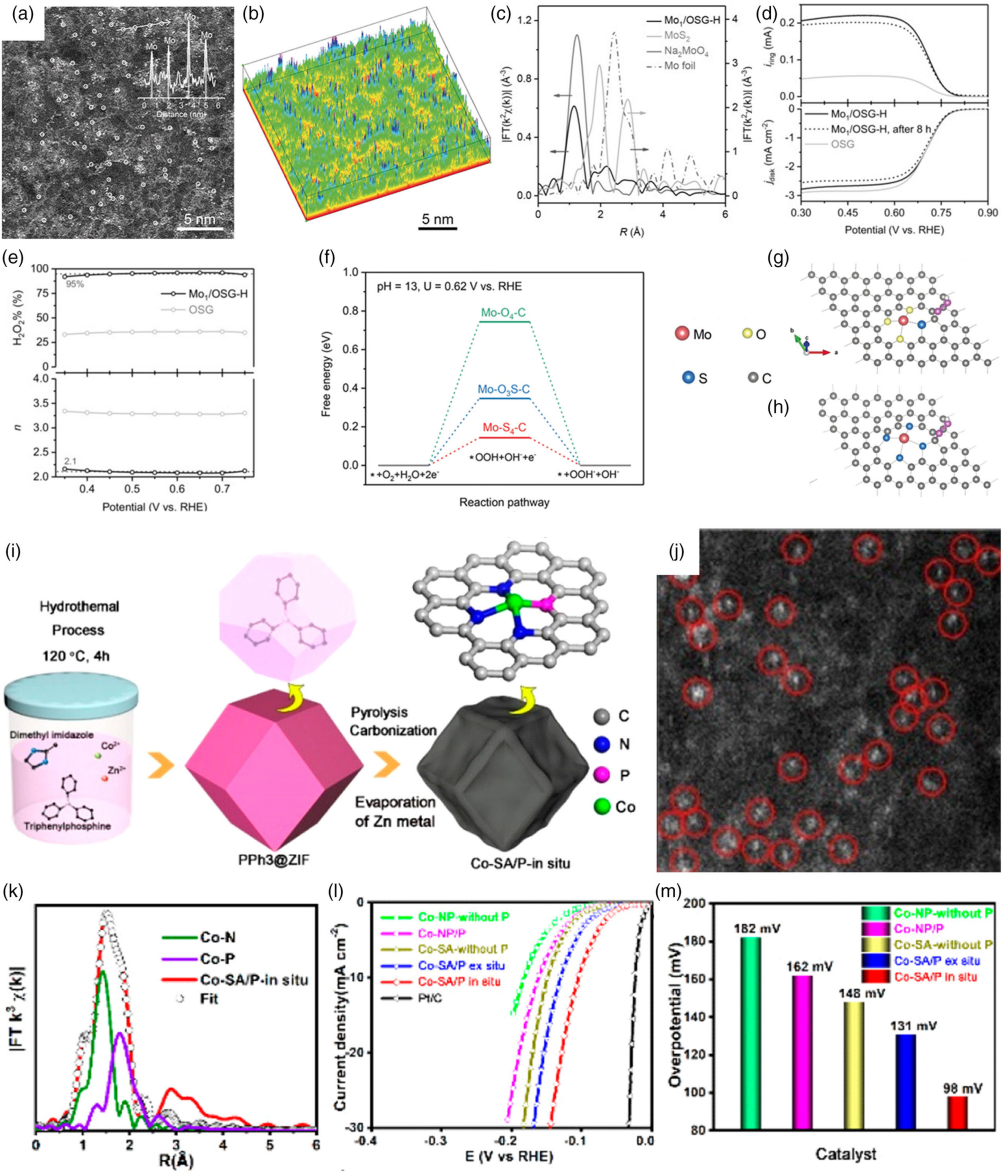
a) HAADF‐STEM image of Mo_1_/OSG‐H with circles marking some single Mo atoms. The inset linear profile shows the intensity across four Mo atoms marked by the arrow. b) Pseudo‐color surface plot corresponding to the HAADF‐STEM image in (a). Blue shows single Mo atoms, red in‐plane pores, yellow defective edges, and green carbon matrix. c) FT‐EXAFS curves of the Mo K edge for Mo_1_/OSG‐H. d) ORR disk current density (*j*_disk_) together with the ring currents (*i*_ring_) at a fixed potential of 1.20 V vs. RHE. The dash line presents the LSV curve after long‐term test and refreshing the electrolyte. e) Calculated electron transfer number (*n*) and H_2_O_2_ selectivity. f) Free energy diagram of 2 e^−^ ORR on three investigated substrates at equilibrium potential of the reaction. g) Atomic configuration of OOH* adsorption on Mo‐O_3_S‐C. h) Atomic configuration of OOH* adsorption on Mo‐S_4_‐C. a–h) Reproduced with permission.^[67d]^ Copyright 2020, Wiley‐VCH. i) Scheme of the formation of Co‐SA/*P*‐in situ. j) Aberration‐corrected STEM images of Co‐SA/*P*‐in situ. k) *k* ^3^‐weight FT‐EXAFS fitting curves of Co‐SA/*P*‐in situ at Co K‐edge. l) HER polarization curves for Co‐SA/*P*‐in situ and other compared catalysts. m) Overpotentials of Co‐SA/*P*‐in situ and other compared catalysts. i–m) Reproduced with permission.^[67e]^ Copyright 2020, American Chemical Society.

For example, Qiao et al. used MgO as a template, and the mixed precursor of carbon source/metal salt/ligand source was pyrolyzed at high temperature to obtain a mesoporous graphene‐supported single‐atom catalyst, and finally a Mo SAC with a unique O,S coordination catalyst was obtained.^[67d]^ Spherical aberration electron microscopy and synchrotron‐based XAS confirmed the local structure and coordination environment of Mo SACs, and clearly identified their unique oxygen‐sulfur double coordination structure. The HAADF‐STEM image and EXAFS further demonstrated isolated Mo atoms were uniformly dispersed on sulfur‐doped graphene (OSG) supports with atomic configurations, suggesting the presence of a single Mo atom (**Figure** **14**a–c). Unlike conventional SACs, this catalyst exhibited a distinct 2‐electron pathway for the ORR reaction in 0.10 m KOH, with a selectivity of up to 95% for hydrogen peroxide (Figure 14d,e). Theoretical calculations showed that changes in the coordination environment (such as increasing the ratio of sulfur) can significantly alter the adsorption capacity of the central metal atom Mo for OOH*, thereby regulating the activity and selectivity of the ORR reaction (Figure 14f–h). This work provided new ideas and strategies for the rational design, synthesis, and regulation of SACs, and also pointed out a direction for the optimization of electrocatalytic reaction selectivity. Recently, Li et al. designed a simple yet novel avenue to develop a single Co atoms catalyst with Co_1_–P_1_N_3_ via in situ phosphatizing of triphenylphosphine that Co species were anchored on N,P co‐doped carbon networks (denoted as Co‐SA/*P*‐in situ) (Figure 14i).[67e] The high‐resolution HAADF‐STEM images and EXAFS further revealed that the N,P co‐doped carbon matrix supported isolated Co atoms were clearly observed and was coordinated by one P atom and three N atoms (denoted as Co_1_P_1_N_3_), and no aggregated Co nanoparticles or clusters were detected (Figure 14j,k). The HER performance demonstrated that the Co‐SA/*P*‐in situ showed a greatly enhanced catalytic performance over the other counterparts and only needed an overpotential of 98 mV to achieve 10 mA cm^−2^ current density (Figure 14l). Furthermore, the Co‐SA/*P*‐in situ exhibited the best activity for HER at 10 mA cm^−2^, compared to those of Co‐SA/*P*‐ex situ (131 mV), Co‐SA‐without P (148 mV), Co‐NP/P (162 mV), and Co‐NP‐without P (182 mV) (Figure 14m). In general, the energy required for the reaction can be better enhanced by adjusting the coordination structure of the metal central atom, thereby improving the catalytic performance.

In general, atomically dispersed catalysts have isolated metal atoms as active sites, anchored by the coordination sites of the surrounding solid support. There are strong interactions or considerable charge transfer between single‐atom metals and supports, and a single species of metal atoms and supports coordinate to prevent atomic diffusion of a single metal atom from agglomerating into particles, relative to the detrimental effects typically suffered by multi‐metallic sites side reactions are highly resistant. Due to their unique structural properties and fully exposed active sites, atomically dispersed catalysts exhibited significantly enhanced catalytic activity in various reactions compared to nanocatalysts. Similar to homogeneous catalysts, atomically dispersed catalysts possessed highly uniform active sites and geometric configurations, which enabled them to share similar electronic structures and steric interactions with substrate molecules, thereby enhancing catalytic selectivity. Furthermore, the spatial separation of metal active sites can effectively suppress unwanted side reactions that occur on multi‐metallic sites.

## 6 Conclusion and Outlook

With the development of advanced characterization techniques and theoretical calculations, the researchers have a deep and intuitive understanding of the relationship between the size and catalytic properties of materials. In addition, researchers have made great breakthroughs in the theoretical basis, synthesis, and catalytic applications of nanometers, sub‐nano, clusters, and atomically dispersed materials. In this review, we have systematically discussed the various catalytic applications of multiscale materials with unique effects, such as size effect confinement effect, defect effect, strain effect, metal–support interactions effect, and coordination effect. 1) NMs have made great progress in the field of preparation and catalysis. However, the current variety of NMs faced many challenges: a) rationally designing NMs composites in a highly controlled manner to achieve excellent performance for specific applications; b) controllably fabricating NMs with different exposed faces is of great significance for regulating their catalytic activity; c) developing facile methods to scale up the yield of NMs to meet the standards for industrial applications. 2) Future work in the field should focus on these issues and promotes the industrial application of clusters. Though the great efforts have been made in this research field, challenges remained to be solved: a) a major issue that needs to be solved is to improve the stability and avoid the deactivation of clusters with high surface energy for catalytic application; b) accurately characterizing and evaluating the dominant active sites of clusters under reaction condition are essential for understanding and further developing advanced cluster catalysts. 3) Opportunities and challenges for atomic‐level catalytic materials: a) it is necessary to explore new methods to synthesize and prepare novel SACs, to study the novel physical and chemical properties of SACs on a larger scale, and to explore their possible applications; b) it is essential to increase the loading of single‐atom catalytic materials for meeting the needs of industrial applications; c) the single‐atom catalysts are used as a model system to observe and track the reaction process with the help of in situ electron microscopy or in situ synchrotron radiation and other in situ characterization methods, and then combine theoretical calculations to study the structure–activity relationship between its structure and catalytic performance, and further explore possible catalytic reaction mechanism; d) since the local electronic structure has a great influence on the performance of materials, it is also very important to precisely control the coordination environment, such as regulating the coordination number and coordination atoms of the active center.

## Conflict of Interest

The authors declare no conflict of interest.
